# Construction and optimization of deep learning intent classification model for smart home dialogue

**DOI:** 10.1371/journal.pone.0357708

**Published:** 2026-09-17

**Authors:** Qiyuan Lu

**Affiliations:** Art and Design School, Lanzhou City University, Lanzhou, China; Philadelphia University, JORDAN

## Abstract

In response to the lack of high-quality annotated corpora in the current smart home field and the insufficient accuracy and interpretability of existing intent classification models in complex home scenarios, this study proposes an intent classification model system that integrates unsupervised topic discovery and deep learning classification. Firstly, a Chinese smart home corpus containing 120,000 real conversations with a dual-layer annotation structure is constructed, and an unsupervised topic recognition model combining latent Dirichlet allocation and K-Means clustering algorithm is designed to mine semantic structures from a data-driven perspective. Finally, a deep learning classification model is designed that integrates a bidirectional transformer model with a text convolutional neural network to achieve accurate intent classification. The unsupervised topic model was superior to the single model in terms of normalized mutual information and adjusted Rand index reaching 0.752 and 0.698 respectively, which verified the rationality of the semantic structure. In terms of supervised learning, the accuracy of the bidirectional transformer-text convolutional neural network model was 96.7%, and the macro F1 score was 96.4%, both of which were better than the baseline model, especially in tasks such as “light control” (F1 score = 98.2%) and “temperature query” (F1 score = 97.6%). Intent distribution analysis further indicated that “device control” (31.2%) and “status query” (24.2%) were the most frequent user needs. Overall, the research provides high-performance intent classification solutions, and also reveals user behavior characteristics at the data level, providing important basis for optimizing the functionality and enhancing the interactive experience of smart home systems.

## 1 Background

With the deep integration of the Internet of Things and artificial intelligence technology, smart home systems are gradually becoming an important component of modern life. Users hope to easily control home devices, query status, and set scenarios through natural language dialogue, which puts extremely high demands on the intent understanding ability of smart home systems [1]. As the core task of natural language understanding, intent classification directly determines the experience and efficiency of human-computer interaction. In the field of intent classification research, there have been numerous studies dedicated to improving model performance. Firdaus M et al. proposed a multi-task model based on Bidirectional Long Short-term Memory Network (BiLSTM) and attention, which synchronously completed dialogue behavior classification, intent detection, and slot filling. The results on the three major publicly available corpora of ATIS, TRAINS, and FRAMES showed that the attention multi-task method consistently outperformed single task and pipeline strong baselines. The dialogue behavior classification improved by over 2%, the intent detection improved by over 1% on ATIS, and by over 3% on TRAINS and FRAMES. The slot filling also improved by 0.8% on ATIS [2]. Das D et al. introduced explainable artificial intelligence (XAI) into smart homes and proposed a framework for explainable behavior recognition. In the remote care scenario, a two-step experiment showed that 92% of the explanations generated by the research method were judged reasonable by experts, and 83% of ordinary users were also willing to read the explanations rather than just the labels [3]. Weld H et al. systematically outlined the evolution of “intent classification+slot filling” from independent training to joint modeling. Firstly, the traditional pipeline was used to solve two tasks separately, and then the shared encoding-multi-task framework, attention, sequence to sequence techniques were used to output intent and slot at once to capture the strong coupling relationship between the two and improve overall accuracy. The study summarized commonly used datasets, evaluation metrics, and experimental designs, and provided performance comparisons of various methods on public benchmarks. The results showed that the joint model generally outperformed the independent model [4]. Zhang H et al. proposed an “adaptive spherical decision boundary” processing method to balance “known intent classification accuracy” and “unknown intent detection” in open intent classification. On three publicly available benchmark datasets, the results showed that this method performed the best [5]. Shang X et al. proposed a collaborative optimization control method for whole vehicle starting based on driving intent classification to address the significant differences in starting performance of heavy-duty vehicles under different starting conditions. Simulation and real vehicle testing showed that this scheme could output the optimal combination of control parameters in real-time according to the recognized intent, significantly shorten the starting time, reduce impact and smoke, and achieve intelligent collaborative control of “how people want to start, the car cooperates optimally” [6].

However, despite the continuous evolution of technology, the research on intent classification for smart home scenarios still faces two key challenges. One reason is the scarcity of domain language materials. The existing public dialogue datasets, such as ATIS, SNIPS, etc., are mostly focused on general domains and lack coverage of the unique intents and expressions of smart homes, resulting in poor adaptability. Secondly, the interpretability of the model is insufficient. Most deep learning models act as “black boxes” and their decision-making processes are difficult to explain, which hinders developers from understanding the performance flaws of the model in specific intents, and also makes it difficult to extract user needs from recognition results to guide product optimization.

Based on this, this study aims to address the following two core research questions: (1) how to build a large-scale high-quality corpus for the field of smart home and establish a reasonable intention tagging system to solve the problem of scarcity of domain corpus? (2) How to design an intention recognition model with high accuracy and interpretability, so that it not only outperforms the existing methods in classification performance, but also reveals the semantic structure of user intention from a data-driven perspective?

For the first research question, this study constructed a Chinese smart home corpus containing 120000 real conversations. The corpus uses a two-tier intention tagging framework (8 main categories and 42 subcategories) that fits the characteristics of the domain, which solves the problem of data missing from the source and provides a reliable basis for subsequent model training and evaluation. For the second research problem, this study uses the two-stage model architecture of “unsupervised topic discovery+supervised deep classification” to solve it. Firstly, the unsupervised topic recognition model is constructed by combining latent Dirichlet allocation (LDA) and K-means clustering (K-means) algorithm. From the perspective of data driven, the hidden semantic structure in the corpus is automatically mined, which is verified with the manual annotation system, so as to enhance the interpretability of the model; Secondly, a supervised intention classification model is constructed by fusing bidirectional encoder representations from transformers (BERT) and text convolutional neural network (textcnn), aiming to achieve high-precision intention recognition. Through the correspondence between the above two research questions and the corresponding methodological components one by one, the research systematically responds to the dual challenges of the scarcity of domain corpus and the lack of model interpretability.

The research aims to systematically enhance the understanding ability of smart home dialogue systems by constructing domain specific corpora and designing high-performance interpretable intent recognition models. The innovation of the research lies in: (1) In theory, a two-stage intention recognition framework that integrates unsupervised topic discovery and supervised classification is proposed. Potential Dirichlet allocation and K-means clustering algorithm are introduced into smart home dialogue semantic mining, and the rationality of the intention annotation system is verified from a data-driven perspective. (2) Technically, a Chinese smart home corpus containing 120000 real conversations with a dual layer annotation structure was constructed, and a bidirectional transformer text convolutional neural network fusion model was designed, achieving a recognition accuracy of 96.7%. (3) In terms of application, quantitative analysis of user intention distribution from the perspective of data mining reveals high-frequency demand patterns centered on “device control” and “status query,” providing practical data support for product function optimization.

## 2 Methods and materials

### 2.1 Construction of a smart home corpus based on real user conversations

**Fig 1:**
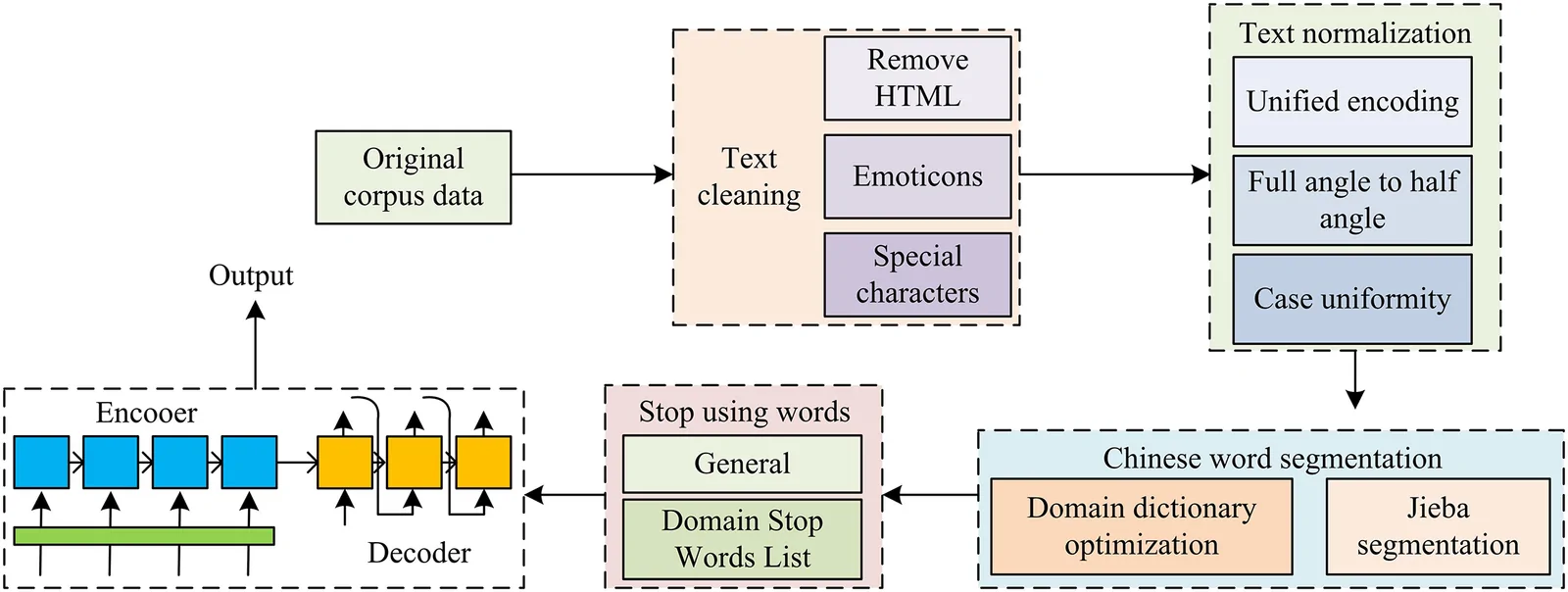
Data preprocessing process.

With the rapid development of IoT, smart homes have become a crucial component of modern homes. Smart home systems can provide users with a convenient device control experience through natural language interaction, and high-quality domain corpora are the foundation for providing good intent classification. Currently, there is a lack of large-scale annotated corpora specifically designed for smart home scenarios, and existing general-purpose dialogue datasets struggle to fully capture the unique linguistic patterns and intent structures inherent to this domain. To address this issue, a corpus in the field of smart homes based on real user conversations is developed. The study collects raw dialogue data from multiple channels for this corpus. The channels for collecting raw dialogue data mainly include user Q&A from Baidu smart home section, desensitization dialogue logs from Tmall Genie devices, discussion posts on smart home forums, and simulated user dialogue data. The initial collection of raw corpus consists of approximately 120,000 dialogue sentences, covering various types of interactions such as device control, status queries, and scene settings. Based on these data, the authenticity and diversity of the corpus can be ensured, which can better reflect the user expression habits in practical application scenarios [7]. In the data preprocessing stage, the study adopts a standardized cleaning process, as shown in Fig 1.

From Fig 1, the rule-based method is first used to remove irrelevant characters, special symbols, and duplicate text, including removing HTML tags, emoticons, and non-textual content [8]. Next, the text normalization processing is performed, including unifying encoding formats, converting full width characters to half width, and unifying English capitalization. In addition, based on the characteristics of Chinese text, the study adopts jieba word segmentation tool for vocabulary segmentation, and optimizes the segmentation effect based on domain dictionaries to ensure that domain terms such as device names and room locations can be correctly segmented [9]. On the basis of word segmentation, the study conducts stop word processing. Specifically, in addition to using a universal stop word list, the research should focus on building a specialized stop word list for the smart home field, removing common but lacking semantic value words such as “please,” “that,” and “this.” Meanwhile, informative vocabulary such as numbers and time words that are important for understanding intent are retained. To further improve data quality, the study also conducts spelling correction and text completion, using sequence to sequence (Seq2Seq)-based error correction algorithms to address common pinyin errors and spelling problems [10]. Based on the above preprocessing process, approximately 850,000 high-quality Chinese word segmentation results are ultimately obtained.

**Fig 2:**
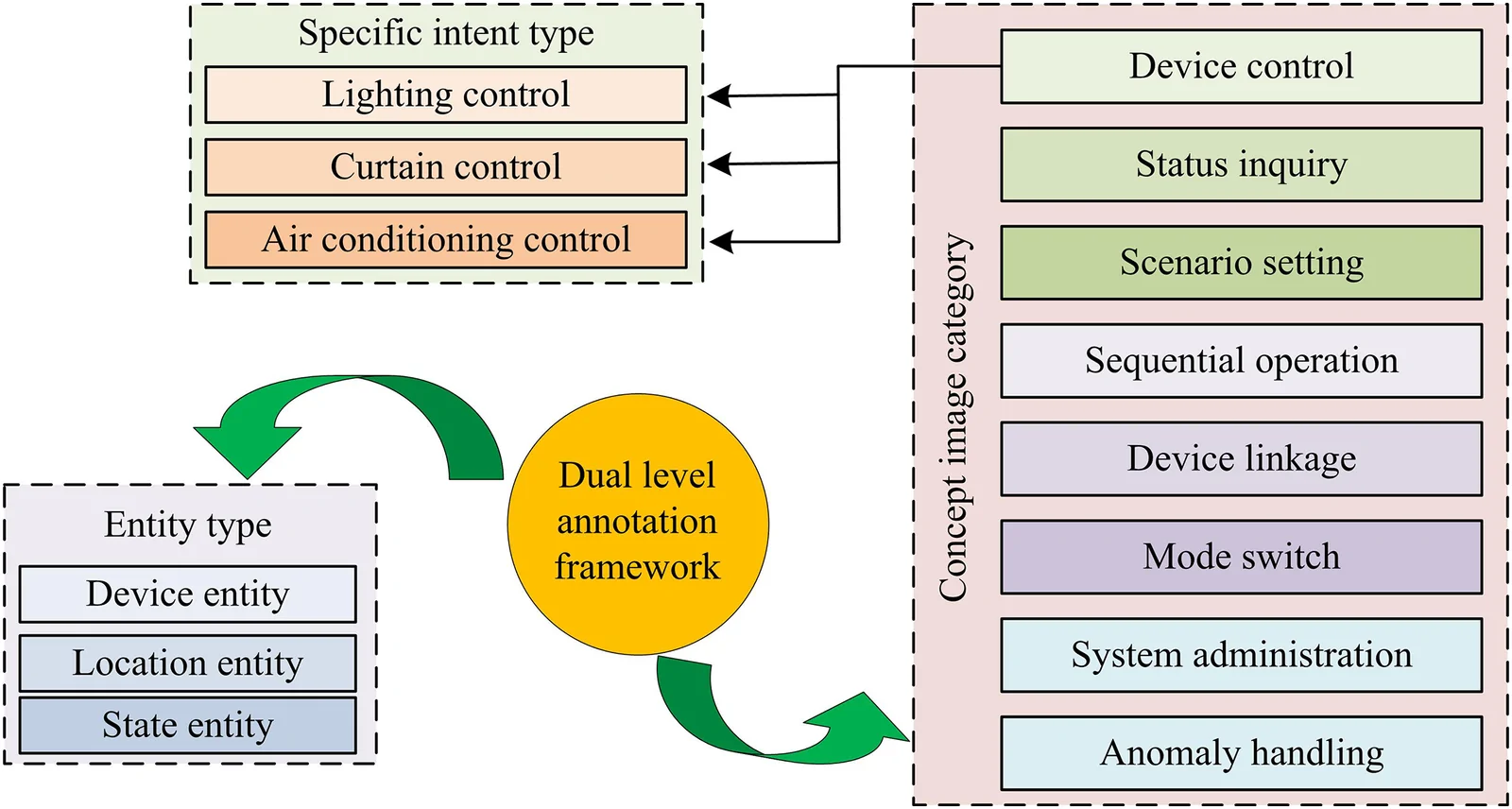
Dual-layer annotation framework.

Although the preprocessed corpus has completed basic text cleaning and standardization, the semantic content still needs to be further structured and annotated to successfully establish a mapping relationship between the corpus and intent categories. Considering the clear intent hierarchy of user queries in the smart home field, which includes both macro functional categories and specific operational instructions, this study proposes to construct a dual-layer annotation framework, as shown in Fig 2.

Fig 2 shows a dual-layer annotation framework. It can be seen that the first layer consists of 8 categories, including device control, status query, scene setup, timing operation, device linkage, mode switching, system management, and anomaly handling. The second layer further refines 42 specific intent types, such as lighting control, curtain control, air conditioning control, etc., under device control. Meanwhile, 9 types of entity annotation standards have been defined, including device entities, location entities, status entities, etc [11]. The tagging process consists of a tagging team composed of five trained taggers, using double-blind tagging and cross validation mechanisms. In order to evaluate the tagging consistency, the study randomly selected 10% of the corpus samples (about 8500 dialog sentences) for multiple tagging, and calculated the Fleiss’ kappa coefficient between taggers. The calculation results show that the average Fleiss’ kappa of the first level idea category is 0.847, and the average Fleiss’ kappa of the second level fine-grained intention category is 0.802, both of which are at the “almost completely consistent” level, indicating that the two-tier annotation system has good reliability and operability. Regularly hold labeling seminars to solve ambiguity, and continuously update labeling guidelines to ensure the consistency of labeling [12]. Ultimately, the constructed corpus can provide important data foundation for user intent classification in smart homes.

### 2.2 Construction of unsupervised intent topic recognition model combining LDA and K-Means

**Fig 3:**
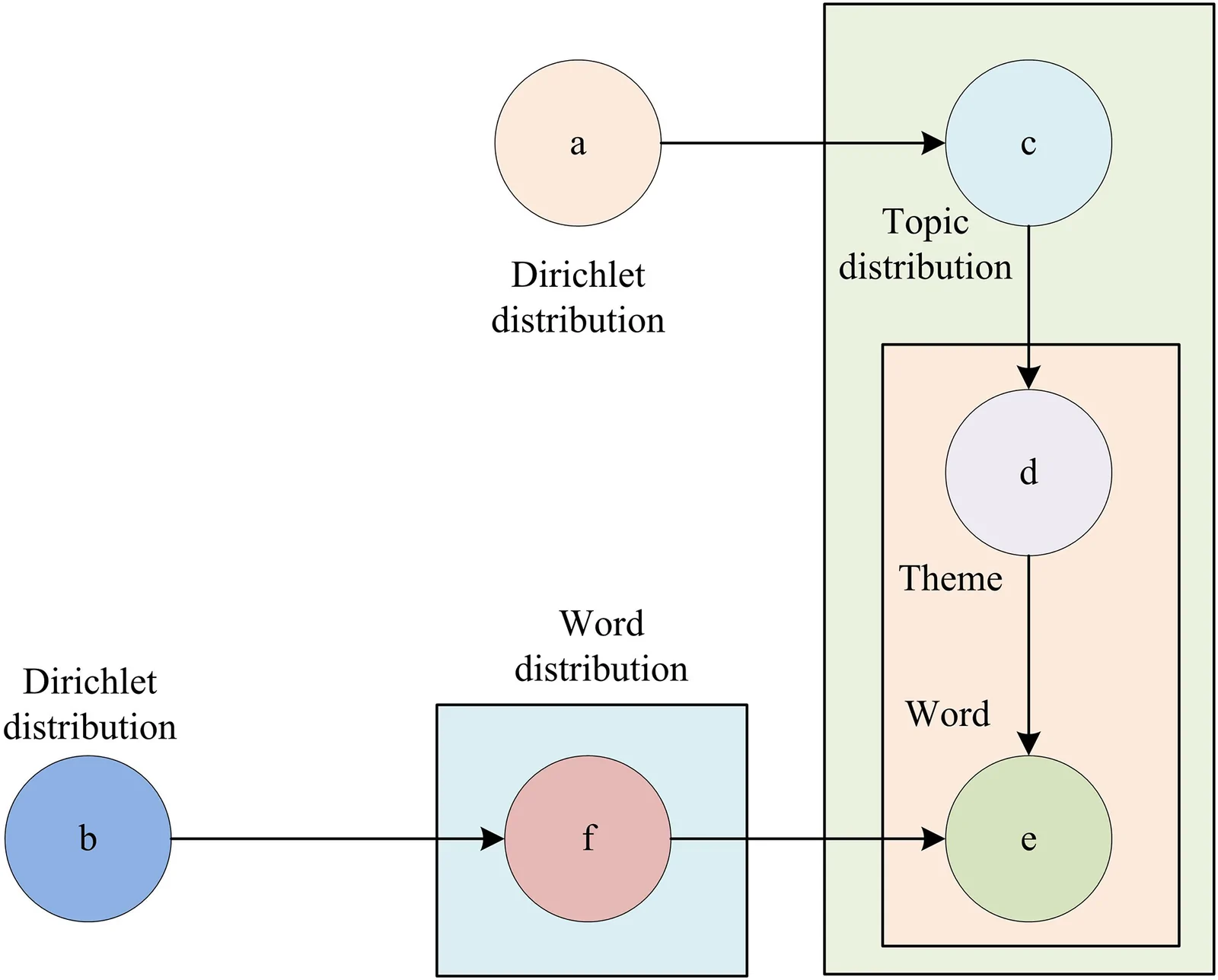
LDA topic model.

After constructing and manually annotating the discourse corpus for smart homes, to verify the rationality of the developed annotation system from a data-driven perspective and deeply explore the underlying semantic topic structure of the corpus, a unsupervised intent topic recognition model is developed by integrating the LDA topic model and K-Means clustering algorithm. This model aims to automatically discover and summarize potential intended topics in dialogue texts without relying on prior annotation information, thereby mutually verifying with the dual-layer annotation framework established based on prior knowledge, and jointly ensuring the scientific and reliable nature of the intent system in both data-driven and knowledge driven dimensions. The model construction first extracts feature words and their probability distributions from the preprocessed dialogue text based on the LDA topic model, as presented in Fig 3.

Fig 3 shows the LDA topic model. LDA is a three-layer Bayesian probability generation model, where each document has multiple topics mixed together, and each topic is a probability distribution on the vocabulary. For each word in the document, a topic is sampled from the document-topic distribution, and then generation words are sampled from the topic-vocabulary distribution. Variational inference estimates model parameters and ultimately obtains the distribution of feature words and their weights in the document for each topic [13,14]. The topic-vocabulary distribution signifies the probability of each word appearing under a topic, while the document-topic distribution represents the proportion of each topic in the document. After obtaining the topic feature word distribution output by the LDA model, these semantic features are further converted into numerical representations that can be used for clustering. In this study, the TF-IDF weights and vectorizes the feature words [15,16]. TF-IDF changes text data into numerical feature vectors by evaluating the importance of a word in a document, as presented in formula (1).


$$\begin{array}{c} \left\{ \begin{array}{c} @l@TF\left(t,d\right)=\frac{n_{t,d}}{\sum_{t^{\prime }\in d}n_{t^{\prime },d}}\\ IDF\left(t,D\right)=log\frac{\left|D\right|}{\left|\left\{d\in D:t\in d\right\}\right|} \end{array}\right. \end{array}$$ (1)

In formula (1), TF(t,d) signifies the word frequency of term t in document d. $n_{t,d}$ signifies the number of times the t appears in d. $\sum_{t^{\prime }\in d}n_{t^{\prime },d}$ signifies the total occurrences of all terms in d [17]. |D| signifies the total documents in the corpus. IDF(t,D) signifies the inverse document frequency of t. |{d∈D:t∈d}| signifies the quantity of documents containing the t [18]. Finally, the TF-IDF value can be expressed as formula (2).


$$\begin{array}{c} TF-IDF\left(t,d,D\right)=TF\left(t,d\right)\times IDF\left(t,D\right) \end{array}$$ (2)

**Fig 4:**
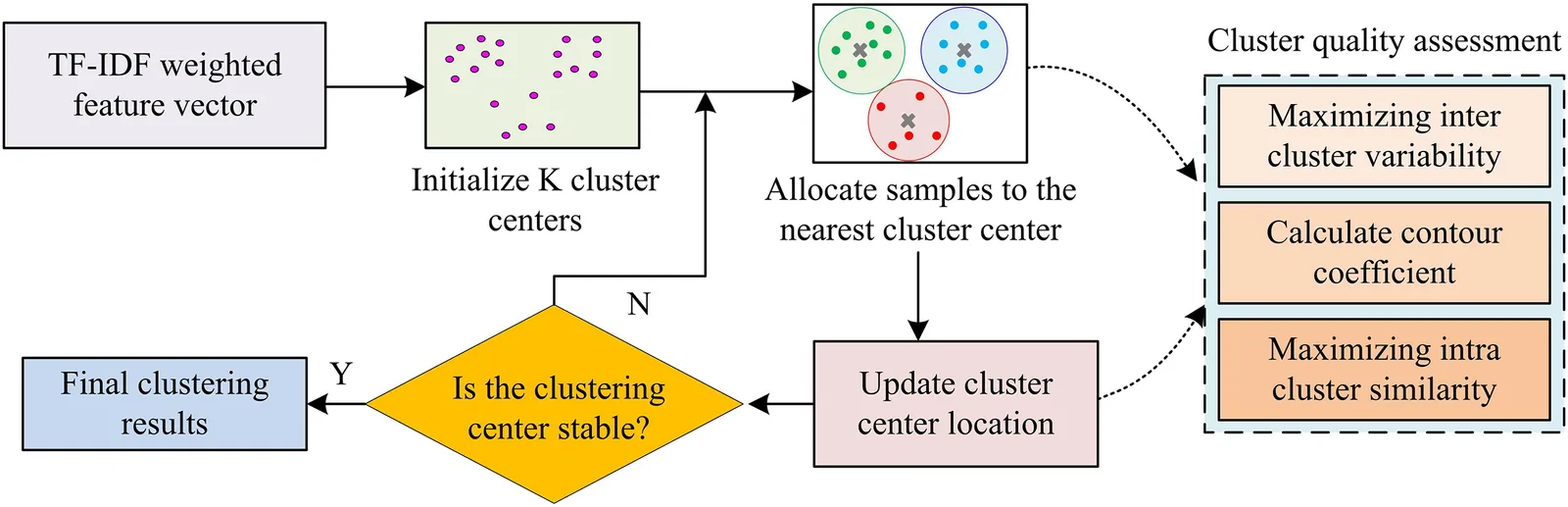
Feature word clustering analysis based on K-Means.

After obtaining the TF-IDF weighted feature word vector representation, to unsupervised discover potential intent topic distribution patterns in smart home conversations, the K-Means is applied to cluster and analyze the feature word vectors. By measuring semantic similarity in the vector space, a set of vocabulary with common semantic attributes is automatically identified and aggregated, revealing the naturally existing intent category structure in the data, as shown in Fig 4.

Fig 4 shows the feature word clustering analysis based on K-Means. K-Means is a distance-based clustering strategy, which aims to divide n samples into k clusters, so that the distance between each sample and its cluster center is minimized [19]. This algorithm iteratively optimizes the cluster center position and sample allocation, thereby maximizing intra cluster sample similarity and inter cluster sample difference. This process can be expressed as formula (3).


$$\begin{array}{c} \left\{ \begin{array}{c} @l@X=\{x_{\text{1}},x_{\text{2}},...,x_{n}\}\\ J=\sum_{i=1}^{k}\sum_{x\in C_{i}}∥x-\mu_{i}{∥}^{\text{2}}\\ \mu_{i}=\frac{\text{1}}{\left|C_{i}\right|}\sum_{x\in C_{i}}x \end{array}\right. \end{array}$$ (3)

In formula (3), X represents the sample set. x is the sample point. J is the objective function. k signifies the preset number of clusters. $C_{i}$ signifies the i-th cluster. $\mu_{i}$ signifies the center point of the i-th cluster [20]. In the clustering process, to determine the optimal number of clusters, the study evaluates the clustering quality by calculating the similarity between the sample and the same cluster or other clusters, as shown in formula (4).


$$\begin{array}{c} s\left(i\right)=\frac{b\left(i\right)-a\left(i\right)}{max\left\{a\left(i\right),b\left(i\right)\right\}} \end{array}$$ (4)

In formula (4), s(i) signifies the contour coefficient of sample point i. a(i) signifies the average distance between i and all other sample points within the same cluster. b(i) signifies the average distance between i and all sample points in the nearest neighbor cluster [21]. Based on the above, extracting semantic features through LDA topic model, combined with TF-IDF weighted vectorization and K-Means clustering analysis, can construct an unsupervised recognition model for intended topics. This joint modeling method not only retains the advantages of LDA in topic discovery, but also utilizes K-Means to optimize the accuracy and interpretability of topic clustering, ultimately achieving unsupervised recognition of dialogue intents in smart homes.

### 2.3 Construction of intent classification model based on BERT-TextCNN

**Fig 5:**
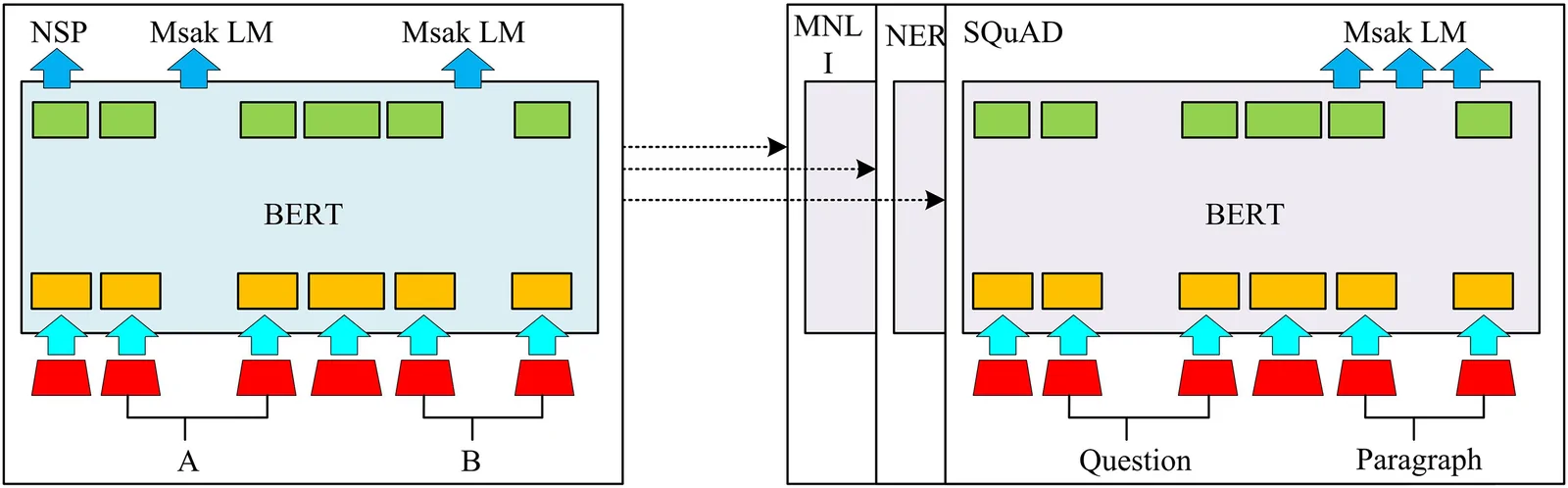
BERT model structure diagram.

After completing the unsupervised exploration of the topic structure of smart home dialogue corpus, a supervised intent classification model that fuses BERT model and TextCNN is developed to further optimize the accuracy and robustness of intent classification. BERT, as a bidirectional pre-trained language model based on Transformer, has shown good performance in multiple natural language processing tasks, as shown in Fig 5.

**Fig 6:**
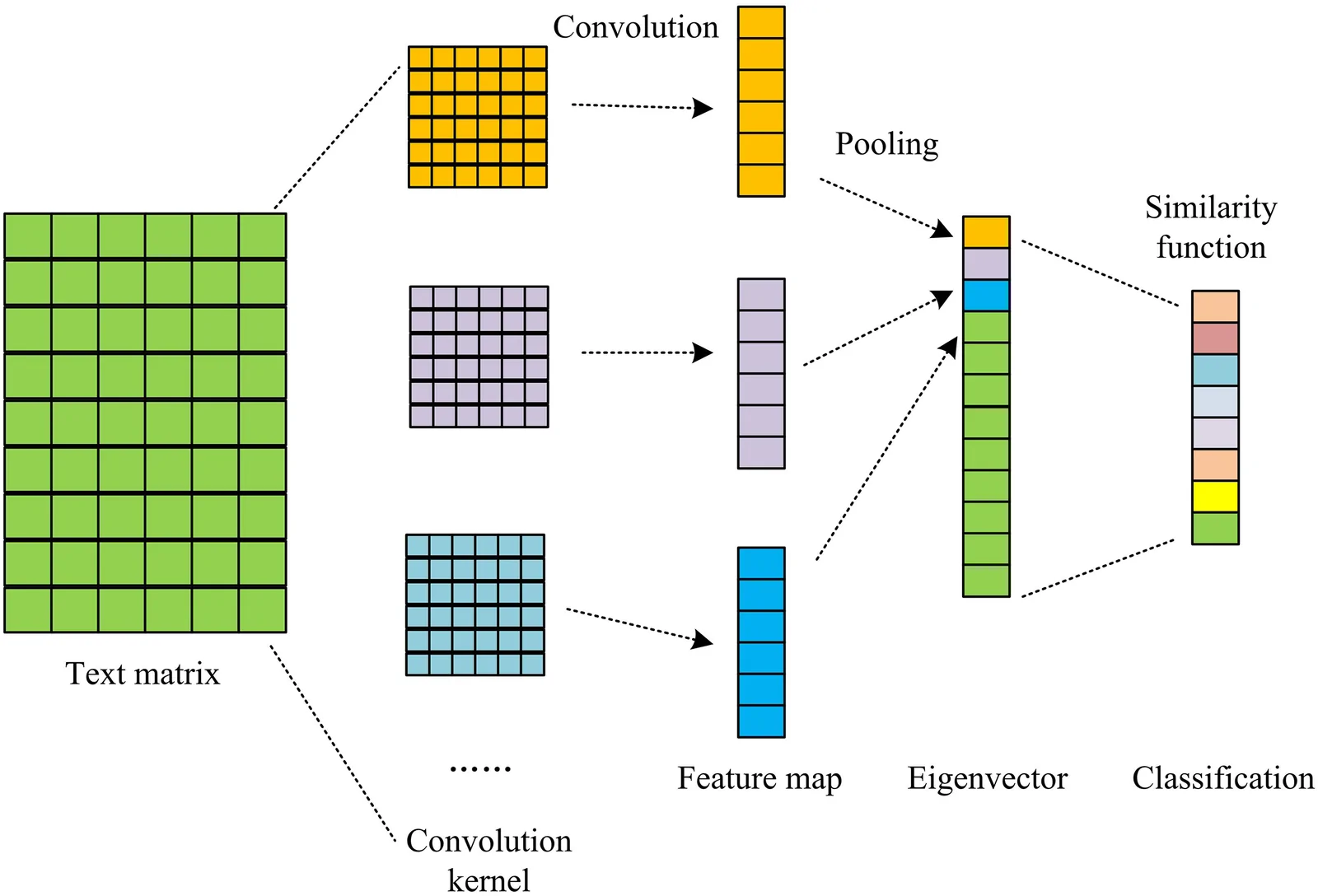
Schematic diagram of TextCNN structure.

Fig 5 is a schematic diagram of the BERT model. BERT captures rich contextual semantic information by pre-training on large-scale corpora through masked language modeling and next sentence prediction tasks. It can be seen that the study uses a BERT-based Chinese pre-trained model as a text encoder to convert the input Chinese dialogue text into a vector representation with semantic information. The input of BERT has word embedding, sentence embedding, and position embedding, which together form the input representation of the model [22]. Considering that the last hidden state of the BERT model contains the richest semantic information and contextual representation ability, which can provide high-quality text representation for downstream tasks, this study adopts a feature extraction strategy based on the last hidden state [23]. Specifically, the hidden states corresponding to the output labels of the last layer of BERT are used as sentence level semantic representations, while the hidden states of other positions in the sequence are used as word level semantic features. To further enhance the ability to capture local semantic patterns, a TextCNN structure is introduced based on BERT output, as shown in Fig 6.

Fig 6 is the structure of TextCNN. TextCNN extracts n-gram features from text through multi-scale convolutional kernels, and filters the most representative features through max pooling operation, ultimately completing classification through the Fully Connected Layer (FCL). To meet the input requirements of TextCNN, the word level semantic feature tensor is transformed into a three-dimensional tensor that conforms to the input format of convolutional neural networks. The first dimension signifies batch size, the second dimension signifies sequence length, and the third dimension signifies feature dimension [24]. The TextCNN module employs a multi-scale convolutional kernel structure to extract local semantic features, and its convolution operation is presented in formula (5).


$$\begin{array}{c} \left\{ \begin{array}{c} @l@K=\{k_{\text{1}},k_{\text{2}},\ldots ,k_{m}\}\\ C_{k}=f(W_{k}*H^{\prime }+b_{k}) \end{array}\right. \end{array}$$ (5)

In formula (5), K signifies the set of convolution kernel widths. k signifies the width of each convolution kernel. K signifies the output feature map of the k-th size convolution kernel. $W_{k}$ signifies the weight matrix of the k-th size convolution kernel. $H^{\prime }$ signifies the input feature tensor. $H^{\prime }$ signifies the bias term for the k-th size convolution kernel. f signifies the activation function [25]. After the convolution output undergoes global max pooling operation, the feature vector is presented in formula (6).


$$\begin{array}{c} p_{k}={max}_{1\leq i\leq L-k}C_{k}[:,i] \end{array}$$ (6)

In formula (6), f is the feature vector of the k-th size convolution kernel after pooling. $C_{k}[:,i]$ represents all channel values in column $C_{k}$ of feature map $C_{k}$. L−k is the sequence length after pooling operation [26]. The final feature representation can be formed by concatenating the feature vectors extracted by all convolution kernels, as shown in formula (7).


$$\begin{array}{c} z=\text{Concat}(p_{\text{1}},p_{\text{2}},\ldots ,p_{m},h_{\text{1}}) \end{array}$$ (7)

In formula (7), z is the final feature vector after concatenation. z is the pooled feature vector corresponding to m convolutional kernel sizes. $h_{\text{1}}$ is the sentence level representation vector corresponding to the label output by BERT [27]. Finally, the feature vector z is input into the FCL. Through the Softmax function, the probability distribution of the intended category can be output, as shown in formula (8).


$$\begin{array}{c} P\left(y|x\right)=\text{Softmax}(W_{z}z+b_{z}) \end{array}$$ (8)

In formula (8), P(y|x) signifies the probability distribution of input x belonging to category y. $W_{z}$ signifies the weight matrix of the FCL. $b_{z}$ signifies the bias term of the FCL. To further ensure the better generalization ability, a Dropout mechanism is introduced before the FCL, which uses random dropout of some neurons to alleviate overfitting. Among them, the model training adopts the cross-entropy loss function, as presented in formula (9).


$$\begin{array}{c} ℒ=-\frac{\text{1}}{N}\sum_{i=1}^{N}\sum_{j=1}^{C}y_{ij}log\left({\hat{y}}_{ij}\right) \end{array}$$ (9)

In formula (9), ${\hat{y}}_{ij}$ is the cross entropy loss. N signifies the number of training samples. C signifies the total intent categories. $y_{ij}$ refers to the true label of sample i. ${\hat{y}}_{ij}$ signifies the prediction probability of the model that sample i belongs to category j [28]. AdamW optimizer is used for parameter updates, and a learning rate decay strategy is taken to improve training stability. Based on the above, the intent classification model can be constructed to classify user intent in smart home scenarios.

## 3 Results

### 3.1 Experimental settings

The experiment uses the smart home discourse database constructed in Section 2.1, which contains 120000 dialogue sentences. According to the ratio of 8:1:1, the data set is randomly divided into training set, validation set and test set. The training set contains 96000 samples, the validation set contains 12000 samples, and the test set contains 12000 samples. The hierarchical sampling strategy is adopted to ensure that the distribution proportion of each intention category in the three subsets is consistent with the original data set. The training set is used for model parameter learning, the validation set is used for super parameter tuning and early stop decision, and the test set is only used for final performance evaluation, and does not participate in any training or tuning process.

All scripts for data preprocessing, model training, evaluation, and figure generation are deposited in the Zenodo repository (https://zenodo.org/records/21998257). The repository provides step‑by‑step instructions to reproduce the dataset splitting, LDA‑KMeans topic analysis, BERT‑TextCNN model training, all evaluation metrics, and visualizations presented in this work. All random seeds were fixed to ensure reproducible experimental outputs.

In the data preprocessing stage, Jieba word segmentation tool is used for Chinese word segmentation, and a stop list containing 196 general stop words and 24 domain specific stop words is constructed at the same time. The maximum length of the text sequence is uniformly set to 128 characters. If it is insufficient, it will be filled in, and if it exceeds, it will be truncated. The LDA topic model sets the number of topics k = 8, uses variational inference to estimate the parameters, sets the training rounds to 20, and sets the number of iterations per round to 200, and uses the automatic learning strategy for the super parameters α and η. The maximum number of TF-IDF feature extraction features is set to 5000, and the word items with document frequency less than 2 or more than 50% are filtered at the same time. The K-means clustering algorithm also sets the number of clusters to 8, uses the k-means++initialization strategy, the maximum number of iterations is 300, and the random seed is set to 42 to ensure that the results can be replicated. In the Bert textcnn model, Bert uses Bert base Chinese pre training weights, with 768 hidden layer dimensions, 12 multiple attention heads, and 12 transformer layers. Textcnn module uses three sizes of convolution cores, which are 2, 3 and 4 respectively. The number of convolution cores of each type is 100, and the convolution is followed by the global maximum pool operation. After the model fusion, the dropout layer is connected, and the discard rate is set to 0.5. Then it is connected to the full connection classification layer, and the output dimension is 42 (corresponding to the number of fine-grained intention categories). Adamw optimizer is used for model training. The initial learning rate is 2 × 10^−5^, the weight attenuation coefficient is 0.01, the batch size is 32, and the training rounds are 20. The learning rate adopts linear warm-up scheduling strategy, and the number of warm-up steps accounts for 10% of the total number of training steps. The loss function is the cross entropy loss, and the maximum norm of gradient clipping is set to 1.0. The verification strategy adopts early stopping method. If the macro average F1 score of the verification set has not been improved for three consecutive rounds, the training will be stopped and the best model weight will be restored. All experiments were run on a single NVIDIA Tesla V100 GPU (32GB video memory), with CUDA version of 11.7, pytorch version of 1.13.0, and transformers library version of 4.25.0.

### 3.2 Model performance comparison and validation

**Fig 7:**
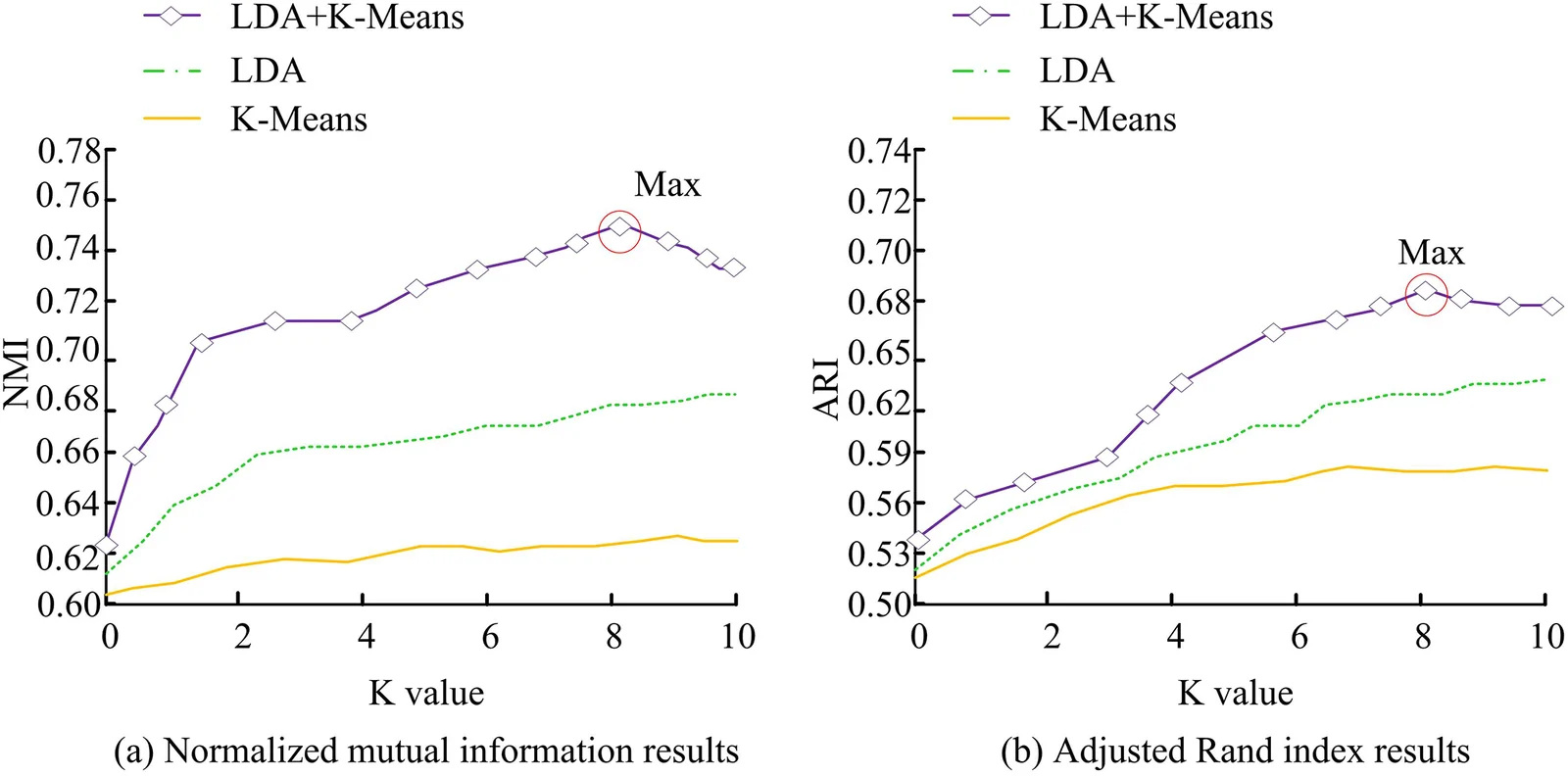
Consistency evaluation between unsupervised topic model and manual annotation.

To comprehensively evaluate the performance of the intent classification model system, the study will be validated from the following two dimensions. Firstly, an internal effectiveness evaluation is conducted on the unsupervised intended topic recognition model that combines LDA and K-Means, to test whether it can discover meaningful semantic structures driven by data itself and mutually verify with the manually constructed dual-layer annotation system. Secondly, the supervised intent classification model based on BERT-TextCNN is compared with various baseline models to verify its accuracy and superiority in the final classification task. All experiments are conducted based on the speech corpus of the smart home constructed in Section 2.1. The consistency evaluation results between the unsupervised topic model and manual annotation are shown in Fig 7.

**Fig 8:**
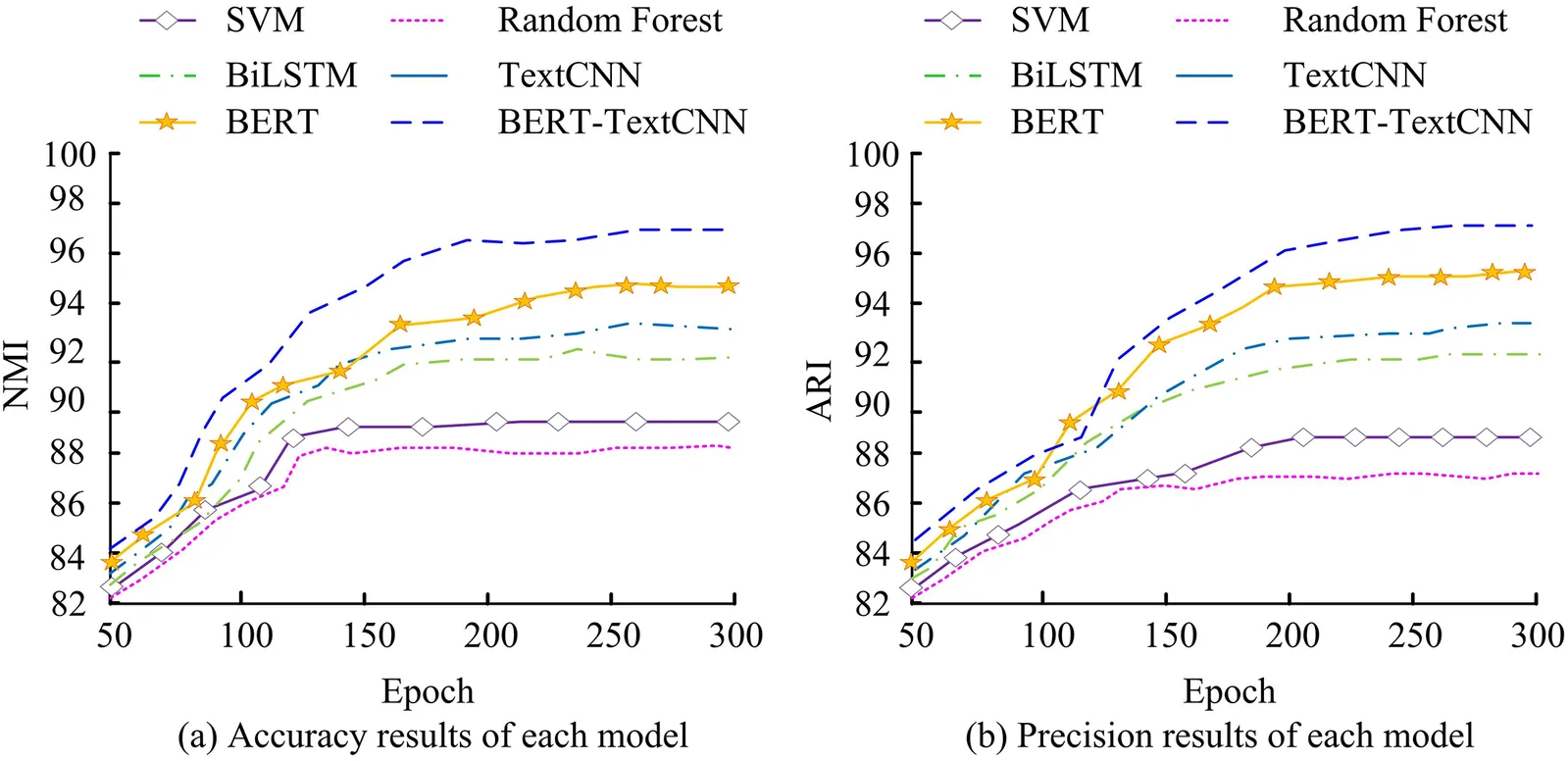
Accuracy and precision results of each model.

Fig 7 shows the consistency evaluation results between the unsupervised topic model and manual annotation. Fig 7(a) shows the Normalized Mutual Information (NMI) results between the output topic and manually annotated categories. The LDA + K-Means recognition model used in the study had a curve trend that first rose, reached a peak near K = 8, and then slowly decreased. This indicates that after extracting features with an appropriate number of topics, the model can most effectively separate the real 8 intent categories. From a specific numerical perspective, the NMI value of the LDA + K-Means recognition model was 0.752, which was better than that of the LDA (0.683) and the K-Means (0.621). Fig 7(b) shows the Adjusted Rand Index (ARI) results between the output topic and manually annotated categories. The ARI of the LDA + K-Means recognition model was 0.698, which was better than that of the LDA (0.635) and the K-Means (0.574). Based on this result, it indicates that there is a strong consensus between the topic clusters automatically discovered by the LDA + K-Means model used in the study and the idea map categories manually annotated, proving the objectivity of the underlying semantic structure of the corpus and the rationality of the manual annotation system. After verifying the effectiveness of the unsupervised model, the experiment further evaluated the supervised intent classification model. The experiment introduced comparative methods, including Support Vector Machine (SVM), Random Forest (RF), and BiLSTM. It is worth noting that all comparison models were trained on the same training set and evaluated for accuracy, precision, recall, and average macro-F1 score on the same testing set. Among them, the results of each model on accuracy and precision are illustrated in Fig 8.

**Fig 9:**
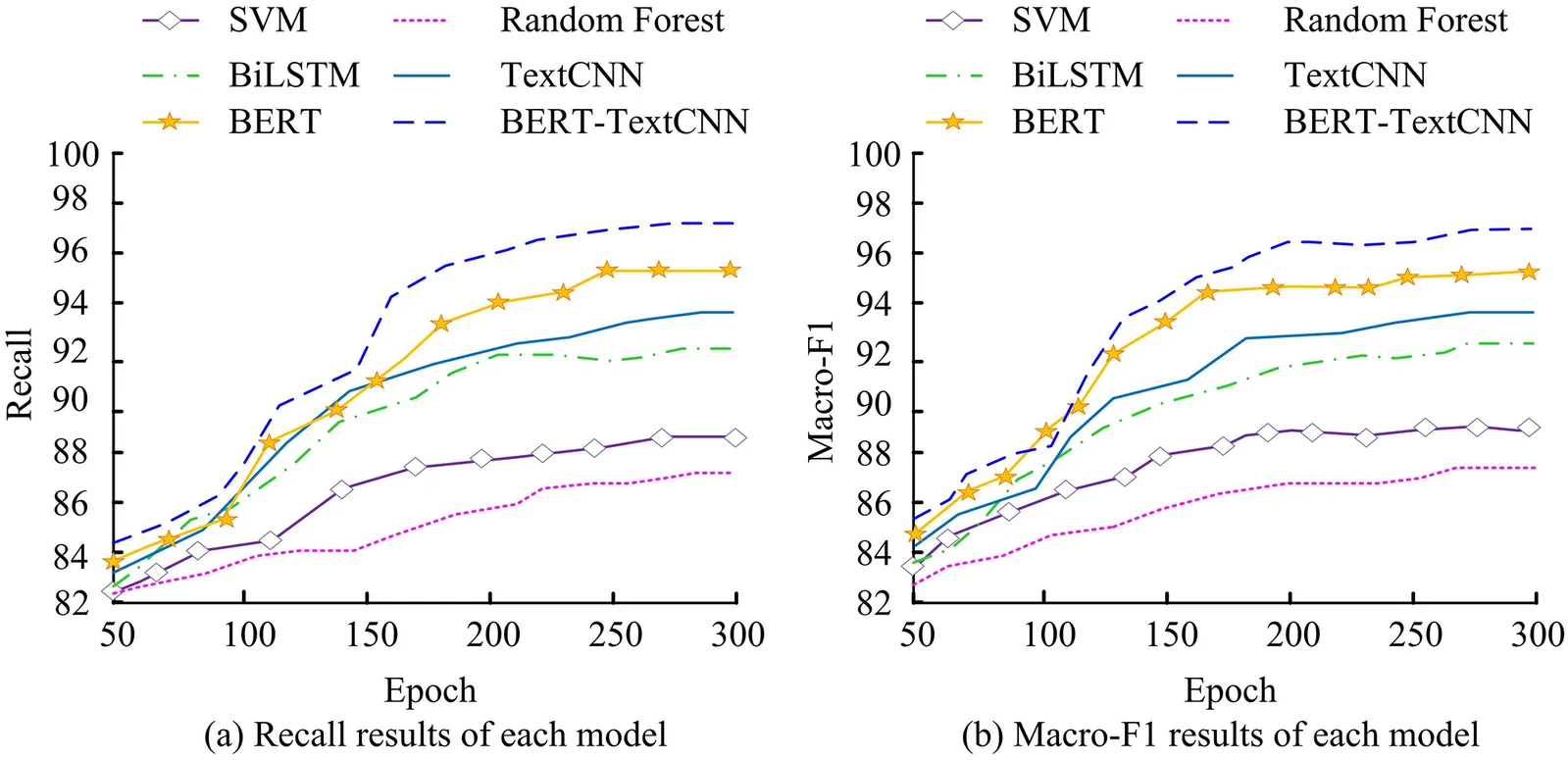
Recall and average macro-F1 score results for each model.

Fig 8 presents the accuracy and precision results. Fig 8(a) presents the accuracy results. The SVM had an accuracy of 89.5% and the RF model had a slightly lower accuracy of 88.1%. In contrast, deep learning models have more obvious advantages, with BiLSTM and TextCNN achieving accuracy of 92.8% and 93.5%, respectively. The BERT model further improved the accuracy to 95.3%. Overall, the BERT-TextCNN fusion model designed in the study had the highest accuracy at 96.7%. Fig 8(b) presents the precision results. The precision of SVM and RF models was 88.7% and 87.9%, respectively. The precision of the BiLSTM model was 92.4%, while the precision of the TextCNN model was 93.2%. The precision of the BERT model was 95.1%. In contrast, the BERT-TextCNN model had the highest precision of 96.5%. In summary, the BERT-TextCNN fusion model significantly outperforms all baseline models in both accuracy and precision, validating its superior performance in intent classification tasks. In addition, the recall rate and average macro-F1 score of each model are illustrated in Fig 9.

Fig 9 presents the recall and average macro-F1 score results. Fig 9(a) presents the recall results of each model. The recall of SVM, RF, BiLSTM, TextCNN, and BERT was 89.6%, 87.8%, 92.6%, 93.3%, and 95.0%. In contrast, the BERT-TextCNN model had the highest recall rate of 96.4%. Fig 9(b) presents the average macro-F1 score results. The Macro-F1 scores of SVM, RF, BiLSTM, TextCNN, and BERT models were 89.2%, 87.8%, 92.5%, 93.2%, and 95.0%, respectively. In contrast, the BERT-TextCNN model had the highest average macro-F1 score at 96.4%. Overall, the BERT-TextCNN model achieves optimal recall and average macro-F1 score, further verifying its comprehensive performance advantages in intent classification tasks. To further analyze the performance on different intent categories, the F1 scores of each model on some representative intent categories were statistically analyzed, as shown in Table 1.

**Table 1:** Details of F1 scores for each model in some intent categories.

Intent Category	SVM	BiLSTM	BERT	BERT-TextCNN
Light Control	90.10%	95.80%	97.50%	98.20%
Temperature Query	91.50%	94.30%	96.80%	97.60%
Scene Setup	85.20%	90.70%	94.10%	95.50%
Anomaly Handling	82.30%	86.50%	90.20%	92.10%

Table 1 shows the F1 scores of each model in some intent categories. In the “light control” task, the F1 score of SVM, BiLSTM, and BERT was 90.1%, 95.8%, and 97.5%. In contrast, the BERT-TextCNN model proposed in the study performed the best, reaching 98.2%. In the “temperature query” task, the SVM model had an F1 score of 91.5%, while the BiLSTM and BERT models had F1 scores of 94.3% and 96.8%, respectively. However, the BERT-TextCNN model still performed the best at 97.6%. For more complex “scene setup” tasks, the performance of each model decreased. The F1 score of the SVM model was 85.2%, while the BiLSTM and BERT models were 90.7% and 94.1%, respectively. In comparison, the BERT-TextCNN model still performed the best at 95.5%. On the most challenging “anomaly handling” task, all models had the lowest performance in the table, with SVM model only achieving 82.3%, BiLSTM and BERT models achieving 86.5% and 90.2%, respectively. However, although BERT-TextCNN model had the lowest numerical performance among all tasks at 92.1%, it still outperformed other models significantly.

To comprehensively evaluate the performance of the model, the study further compares it with a variety of recent advanced methods, including pure Bert classifier, pure textcnn and bilstm+attention mechanism. The selected baseline covers the mainstream technical route from traditional machine learning to pre training model, and can comprehensively test the comparative advantages of the proposed model. The overall performance comparison of each method on the test set is shown in Table 2.

**Table 2:** Comparison with advanced models.

Model	Accuracy	Macro F1
SVM	89.50%	89.20%
BiLSTM + Attention	93.60%	93.10%
TextCNN	93.50%	93.20%
BERT-base	95.30%	95.00%
BERT + BiLSTM + Attention	95.80%	95.50%
BERT-TextCNN	96.70%	96.40%

It can be seen from Table 2 that the proposed model performs best among all comparison methods. Compared with pure Bert, the accuracy of the fusion of textcnn is improved by 1.4%, indicating that the local n-gram feature has supplementary value for intention recognition Compared with bilstm+attention, it increased by 3.1%, which verified the advantages of the pre training model, and also showed that the closed intention classification architecture of this study was more suitable for smart home scenarios. The model is effective. The unsupervised topic model validated the rationality of the semantic structure of the corpus from a data-driven perspective, laying a reliable data foundation for the entire study. Supervised classification models, relying on advanced architecture fusion, have better performance in intent classification tasks, providing a reliable technical solution for natural language interaction in smart homes.

Finally, the research selects the most representative cutting-edge methods for smart home intention recognition task in recent years for comparison. In order to clearly show the performance differences of each model, Table 3 summarizes the key indicators of the above frontier methods and the model proposed in this study in the task of smart home intention recognition.

**Table 3:** Performance comparison between the proposed model and state-of-the-art methods.

Model	Accuracy	F1-Score	Deployment Environment
GloVe-BiGRU-self-attention [29]	89.72%	—	Server-side
Qwen2.5−0.5B (16-bit) [30]	98.80%	—	Edge device
Qwen2.5−0.5B (4-bit quantized) [30]	81.70%	—	Edge device
mBERT+Squeezeformer-CTC [31]	—	91.50%	Resource-constrained device
BERT-TextCNN	96.70%	96.40%	Server-side

Note:”—” indicates that the index is not involved in the quoted literature

It can be seen from Table 3 that although the accuracy (98.8%) of the 16 bit large language model of birkmose et al. [30] is slightly higher than that of this study, the performance of its 4-bit quantized version has dropped sharply to 81.7%, indicating that the generative large language model faces a severe accuracy efficiency tradeoff when deployed at the edge, and the Bert textcnn model in this study avoids the performance loss caused by quantization with a lighter text coding architecture. Compared with the glove bigru self attention model of Li et al. [29], the accuracy of this study increased by nearly 7%, which verified the advantages of the pre training language model in smart home intention recognition. Compared with the 91.5% F1 score obtained by vysotska et al. [31] under the condition of limited resources, the macro average F1 score of this study reached 96.4%, which also showed obvious performance advantages. To sum up, the Bert textcnn model maintains high accuracy and robustness in closed intention classification tasks, and has better deployment flexibility than the generative large language model, which fully verifies its comprehensive advantages in practical applications.

### 3.3 Intent topic distribution and practical application analysis

After verifying the effectiveness of the unsupervised topic model and the accuracy of the supervised classification model, the study further analyzed the deep semantic structure of the LDA topic model, aiming to explore the distribution pattern of potential intent topics in smart home user conversations, and combine the analysis results with practical application scenarios to provide data-driven insights for product optimization and user experience improvement. This analysis is based on the joint LDA and K-Means unsupervised recognition model. The model achieved the best NMI when the number of topics K = 8, and formed a good correspondence with the 8 manually annotated mind map categories. The experiment extracted the top 10 feature words with the highest probability under each topic, and invited three domain experts to summarize and name the core semantics of each topic to connect data-driven topics with knowledge-driven application scenarios. The Top-10 feature words and their probability distributions under each concept topic are presented in Table 4.

**Table 4:** Top-10 feature words and their probability distributions under each topic.

Topic ID	Expert-Annotated Topic Name	Top-10 Feature Words and Their Probabilities
1	Device Control	light(0.118), turn_on(0.095), turn_off(0.087), brighten(0.062), dim(0.054), bedroom(0.041), living_room(0.038), switch(0.035), device(0.032), brightness(0.029)
2	Status Query	temperature(0.121), what(0.094), current(0.082), set(0.071), air_conditioner(0.069), humidity(0.057), query(0.046), how(0.041), report(0.036), status(0.033)
3	Scene Setup	scene(0.142), mode(0.113), activate(0.089), home(0.075), theater(0.064), sleep(0.058), setup(0.051), create(0.043), custom(0.037), enable(0.034)
4	Timing Operation	timer(0.152), minute(0.126), hour_later(0.094), tomorrow(0.081), morning(0.072), schedule(0.063), o'clock(0.057), evening(0.048), turn_off(0.042), remind(0.039)
5	Device Linkage	linkage(0.181), if(0.145), then(0.112), automatic(0.103), trigger(0.088), condition(0.076), execute(0.064), door_lock(0.051), turn_on(0.047), sensor(0.041)
6	Mode Switching	mode(0.164), switch(0.138), energy_saving(0.124), comfort(0.109), cooling(0.096), heating(0.087), turn_on(0.075), turn_off(0.062), air_purification(0.054), fan_speed(0.048)
7	System Management	network(0.171), connect(0.154), password(0.132), reset(0.118), settings(0.104), bluetooth(0.091), device(0.085), name(0.076), update(0.069), system(0.062)
8	Anomaly Handling	error(0.203), what_to_do(0.187), solve(0.165), alarm(0.142), online(0.126), support(0.118), help(0.104), customer_service(0.097), connection_failed(0.088), diagnose(0.081)

**Fig 10:**
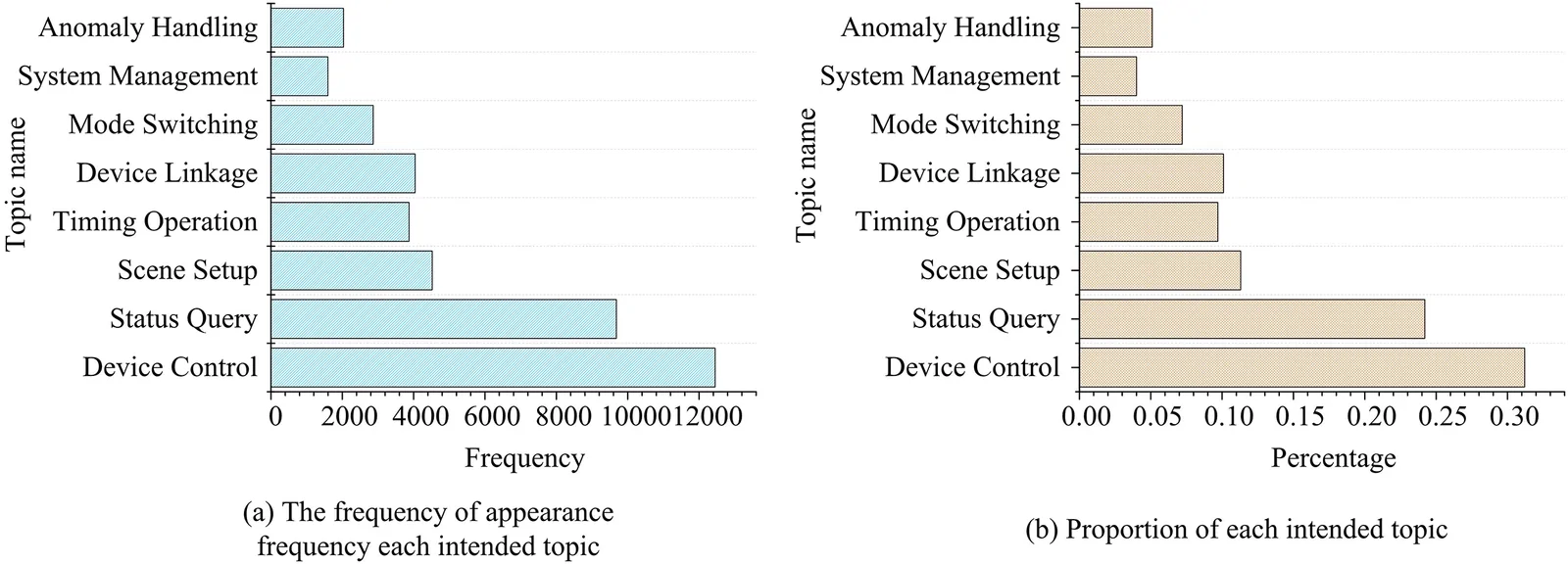
Distribution frequency of each intended topic.

Table 4 presents the Top-10 feature words and their probability distributions for each topic in the mind map. Each topic presents highly concentrated semantic features, with the “device control” topic centered around operations such as “light” (0.118), “turn_on” (0.095), and “turn_off” (0.087). The “status query” is dominated by inquiry vocabulary such as “temperature” (0.121), “what” (0.094), “current” (0.082), etc. The feature words of the “scene setup” include “scene” (0.142), “mode” (0.113), “activate” (0.089) and other scene related terms. The “time sequence operations” mainly includes time expressions such as “timer” (0.152), “minute” (0.126), and “hour_later” (0.094). The “device linkage” is characterized by conditional logic vocabulary such as “linkage” (0.181), “if” (0.145), “then” (0.112), etc. The feature words of the “mode switching” are “mode” (0.164), “switch” (0.138), “energy_saving” (0.124) and other mode related terms. The “system management” includes system level vocabulary such as “network” (0.171), “connect” (0.154), “password” (0.132), etc. The “anomaly handling” mainly focuses on fault handling vocabulary such as “error” (0.203), “what_to_do” (0.187), and “solve” (0.165). These feature words and their probability distributions clearly reflect the semantic core of each intended topic, verifying the effectiveness of unsupervised topic models in discovering domain specific semantic structures. To transform the analysis results of the topic model into practical application value, the study further calculated the distribution ratio of each intent topic in the test set, that is, the frequency of different intents appearing in real user interactions, as shown in Fig 10.

Fig 10 shows the distribution frequency of each intended topic in the test set. Fig 10(a) shows the appearance frequency for each intended topic, with a total frequency of 40,060 occurrences for all topics. Among them, the device control topic had the highest frequency of occurrence, reaching 12,450 occurrences. Next was the status query topic, which appeared 9,683 times. The appearance frequency of the scene setup topic was 4,521 times, the appearance frequency of the device linkage topic was 4,038 times, the appearance frequency of the timing operation topic was 3,872 times, the appearance frequency of the mode switching topic was 2,865 times, the appearance frequency of the anomaly handling topic was 2,035 times, and the appearance frequency of the system management topic was the lowest, at 1,596 times. Fig 10(b) shows the proportion distribution of various intended topics. The device control topic had the highest proportion, reaching 31.2%, the status query topic was 24.2%, the scene setup topic was 11.3%, the device linkage was 10.1%, the timing operation topic was 9.7%, the mode switching topic was 5.1%, and the system management topic was 4.0%. These distribution data clearly reflect the key distribution of user needs in the smart home scenario, providing important basis for system optimization and resource allocation. Based on this result, combined with the recognition accuracy of each intent category mentioned above, a “weighted performance score” can be further calculated. This score can be used to assess the overall performance in real-world applications and provide guidance for optimizing priorities, as presented in Table 5.

**Table 5:** Weighted performance contribution analysis of each intent.

Topic Name	F1 score	Distribution Frequency	Weighted Performance Contribution
Device Control	97.8%	31.2%	30.1%
Status Query	97.5%	24.2%	23.6%
Scene Setup	96.2%	11.3%	10.9%
Timing Operation	95.1%	9.7%	9.2%
Device Linkage	95.8%	10.1%	9.7%
Mode Switching	95.3%	7.2%	6.9%
System Management	93.6%	4.0%	3.7%
Anomaly Handling	92.9%	5.1%	4.7%

Table 5 shows the weighted performance contribution analysis results of each intent. The weighted performance contribution of device control intent was the highest, reaching 30.1%. The status query ranked second with a contribution rate of 23.6%. The contribution of scene setup was 10.9%, the device linkage was 9.7%, the timing operation was 9.2%, the mode switching was 6.9%, the system management was the lowest, only 3.7%, and anomaly handling was 4.7%. The overall performance in real-world scenarios is mainly dominated by high-frequency and high-precision intents. Finally, based on topic feature word analysis, the study recommended the most relevant response strategies and product function optimization directions for each intended topic, as shown in Table 6.

**Table 6:** Recommended application optimization directions based on topic feature words.

Topic Name	Key Feature Word Insights	Recommended Optimization Directions
Device Control	High frequency of location entities (bedroom, living_room)	Develop context-aware control based on user location
Status Query	Multi-dimensional status inquiries (temperature, humidity)	Provide aggregated status report cards supporting multi-condition queries
Timing Operation	Rich time expressions (minute, hour_later, tomorrow morning)	Enhance natural language time parsing capability supporting complex scheduling
Device Linkage	Conditional logic words (if, then, automatic)	Simplify smart rule configuration process with natural language
Mode Switching	Various mode terms (energy_saving, comfort, cooling)	Implement one-click mode switching with voice commands
System Management	System-level operation words (network, connect, reset)	Optimize device connection and network configuration workflow
Anomaly Handling	Strong help-seeking signals (what_to_do, solve, customer_service)	Strengthen self-diagnosis system and seamless customer service transfer

Table 6 shows the recommended application optimization directions based on topic feature words. For high-frequency location entity vocabulary in device control topics, it is recommended to develop context aware control functions based on user location. It is recommended to provide aggregated status report cards that support multi-condition queries for the multi-dimensional status query features in the status query topic. For the rich time expressions in the timing operation topic, it is recommended to enhance the natural language time parsing ability to support complex timing settings. For the conditional logic vocabulary in the device linkage topic, it is recommended to simplify the intelligent rule configuration process through natural language. For various mode terms in the mode switching topic, it is recommended to implement a one click mode switching function that supports voice commands. For the system level operational vocabulary in the system management topic, it is recommended to optimize the workflow of device connection and network configuration. For the strong distress signal vocabulary in the anomaly handling, it is recommended to strengthen the fault self-diagnosis system and seamlessly transfer the customer service process. Overall, these optimization directions are directly derived from data mining of users’ real expressions, which can provide data support for improving the functionality and user experience of smart home systems.

## 4 Discussion

Compared with existing research, the proposed intent classification framework has significant differences and complementary value in task objectives and technical paths. Ding J et al. [32] focused on the dialogue emotion recognition task and pointed out that traditional methods often overlook the interaction between the attitudes and behaviors of both parties in the dialogue. They then designed a DialogueINAB based on the “attitude-behavior” theory. This model utilized three modules of perception, interaction, and classification, combined with a cross-modal Transformer to capture the dynamic correlation between potential attitudes and speech acts of both parties in a conversation. Significant performance improvements have been achieved on multiple datasets such as IEMOCAP, MELD, and AVEC, validating the effectiveness of introducing social psychology mechanisms for understanding conversational emotions. In contrast, the research focuses on user intent classification in smart home scenarios, emphasizing the intent understanding accuracy and interpretability from two aspects: semantic structure and classification accuracy. The two complement each other in task category and methodology. In addition, Torad M A et al. [33] proposed a voice-driven automation system for IoT smart homes, which parses user instructions into executable operations through natural language processing technology, and uses hardware platforms such as Arduino and Raspberry Pi to achieve device control and energy management, while introducing policy recommendation functionality based on machine learning. This study focuses on system integration and practical deployment, while this research pays more attention to the structural innovation and performance optimization of the intent classification model, especially in the in-depth exploration of semantic representation and classification accuracy. Therefore, the contribution of the model architecture and semantic mining level can provide more reliable intent understanding core components for systems such as Torad, with good embedding and expansion potential. In summary, the proposed model that integrates unsupervised topic discovery and supervised classification in the field of smart home intent classification not only differs from sentiment recognition and system integration research in terms of technical path, but also achieves significant improvements in semantic interpretability and classification performance, providing important references for subsequent research and applications in this field. However, due to the fact that the corpus source is mainly Chinese and the coverage of scenarios is limited, the generalization ability of the model may still have certain limitations in multilingual, multi-dialect, and extremely complex interactive scenarios. In the future, further optimization can be achieved by introducing multilingual pre-training models, enhancing adversarial sample training, and constructing larger cross scenario corpora.

To further understand the performance boundary of the model, the samples that were incorrectly classified in the test set were systematically analyzed. Statistics show that the overall error rate of the Bert textcnn model on the test set is 3.3%, and about 62% of the errors occur between intention categories with similar semantics. The most common confusion pairs include “temperature query” misjudged as “humidity query,” “light brightness adjustment” misjudged as “light switch control” and “scene setting” misjudged as “mode switching.” The analysis shows that such errors are mainly due to the lack of clear distinguishing keywords in user expression. Secondly, about 23% of the errors are related to complex sentence patterns, especially queries containing negative words or conditional clauses. The model performs poorly in dealing with such logical dependencies. Third, about 15% of the errors occurred in the intention category with less training samples. For example, the F1 scores of “system management” and “exception handling” were 93.6% and 92.9%, respectively, which were lower than the high-frequency intention category, indicating that the data imbalance had a negative impact on the performance of the model. The above analysis shows that the main failure modes of the current model focus on the differentiation of semantically similar intentions, the understanding of complex logical expressions, and the learning of low-frequency categories.

## 5 Limitations and future work

This study has some limitations and needs to be further improved in future work. First of all, the corpus is mainly from Chinese. Although it covers the mainstream smart home interaction scene, it has not yet included dialect, Chinese English hybrid and multilingual dialogue data. The generalization ability of the model in non-standard Chinese expression still needs to be verified. Secondly, the current intention system is based on the closed world hypothesis, which assumes that all user inputs can be classified into 42 predefined intention categories, and the recognition ability of unknown intentions or new intention categories that do not appear in the training set has not been evaluated. Third, although the corpus contains 120000 conversations, it is mainly collected from public forums, device logs and simulated conversations. It lacks long-term continuous conversation data in a large-scale real home environment, and may not fully reflect the conversation context dependency in actual use. Fourth, the model training and evaluation are carried out on a single GPU server, and its reasoning efficiency has not been tested on edge computing devices or resource constrained embedded systems. The real-time performance of the actual deployment needs to be tested. Finally, the current model only supports intention recognition in a single round of conversation, and has not been extended to complex scenarios such as intention evolution tracking and context slot inheritance in multiple rounds of conversation. In the future, it can be further optimized by introducing a multilingual pre training model, enhancing the training of confrontation samples, building a larger cross scene corpus, and developing lightweight model compression technology.

Looking forward to the future, the research will continue from the following directions. Firstly, in view of the confusion of the current model in the category of semantic similar intentions, it is planned to introduce a fine-grained comparative learning loss function to enhance the ability of the model to distinguish similar intentions. Secondly, aiming at the limitation of the lack of understanding ability of complex sentence patterns, this paper intends to explore the method of combining graph neural network with pre training model to explicitly model syntactic dependencies and logical structures. Third, in order to alleviate the impact of data imbalance on the performance of low-frequency categories, we will study the small sample learning strategy based on cue learning to reduce the dependence of the model on large-scale labeled data. Fourth, the current model only supports single round intention recognition. In the future, it will be extended to multiple rounds of conversation scenarios to develop a context aware intention tracking and slot inheritance module. Fifthly, facing the actual deployment requirements, it is planned to compress the current model through knowledge distillation and model quantification technology, so that it can run efficiently on edge devices such as smart speakers. Finally, a large-scale corpus containing multilingual, multilingual and real family continuous dialogues will be constructed to further enhance the generalization ability and ecological adaptability of the model.

## 6 Conclusion

A complete and efficient deep learning solution was developed for user intent classification in smart home scenarios. The main findings were as follows: (1) The proposed BERT-TextCNN fusion model performed well in intent classification tasks, with accuracy, precision, recall, and average macro-F1 scores all exceeding 96%, which was superior to traditional machine learning and single deep learning models, effectively improving the accuracy and robustness of human-machine interaction in smart homes. (2) The unsupervised topic discovery model Based on LDA and K-Means is proposed, which verified the semantic structure features of smart home conversations at the data level, and discovered the core intent distribution mainly based on “device control” and “status query,” providing clear direction for product optimization. In summary, the study not only proposes a high-performance intent classification model, but also reveals the essence of user needs from the perspective of data mining, providing theoretical and practical support for the development of natural language interaction systems in smart homes. The research results have direct application value for improving home intelligence and user experience.
