# Supplementary material for: Construction and optimization of deep learning intent classification model for smart home dialogue
Source: PLoS One. 2026 Sep 17;21(9):e0357708. doi: 10.1371/journal.pone.0357708 (PMC13585276; doi:10.1371/journal.pone.0357708)
Supplement: Supplementary file 1 [file pone.0357708.s004.docx]

PONE-D-26-09779R2

Construction and Optimization of Deep Learning Intent Classification Model for Smart Home Dialogue

PLOS One

Dear Dr. Lu,

Thank you for submitting your manuscript to PLOS ONE. After careful consideration, we feel that it has merit but does not fully meet PLOS ONE’s publication criteria as it currently stands. Therefore, we invite you to submit a revised version of the manuscript that addresses the points raised during the review process.

==============================

The replication package appears incomplete, as all references currently point to .docx files. Please provide the correct executable/reproducible code and update the associated file references to enable verification of the reported results.

==============================

Reply: The revised data and code have been archived to meet the reproducibility requirements of PLOS ONE. All original data tables and source analysis scripts are now uniformly stored in a Zenodo repository. The code is provided in the form of independent executable script files rather than being embedded in Word documents. All files are publicly accessible, facilitating independent verification of our analysis results. The main text of the paper and this reply letter both reference the same repository.

The data and code have been available on https://zenodo.org/records/21834289
