# Supplementary material for: Construction and optimization of deep learning intent classification model for smart home dialogue
Source: PLoS One. 2026 Sep 17;21(9):e0357708. doi: 10.1371/journal.pone.0357708 (PMC13585276; doi:10.1371/journal.pone.0357708)
Supplement: Supplementary file 2 [file pone.0357708.s002.doc]

Journal Requirements:

When submitting your revision, we need you to address these additional requirements.

1.Please ensure that your manuscript meets PLOS ONE's style requirements, including those for file naming. The PLOS ONE style templates can be found at

https://journals.plos.org/plosone/s/file?id=wjVg/PLOSOne_formatting_sample_main_body.pdf and

https://journals.plos.org/plosone/s/file?id=ba62/PLOSOne_formatting_sample_title_authors_affiliations.pdf

Reply: Thank you very much for your comments. The revised paper complies with the formatting requirements of PLOS ONE.

2. Please note that PLOS One has specific guidelines on code sharing for submissions in which author-generated code underpins the findings in the manuscript. In these cases, we expect all author-generated code to be made available without restrictions upon publication of the work. Please review our guidelines at https://journals.plos.org/plosone/s/materials-and-software-sharing#loc-sharing-code and ensure that your code is shared in a way that follows best practice and facilitates reproducibility and reuse.

Reply: The "Code" file of the manuscript is available at:

https://zenodo.org/records/20644845

3. We note that your Data Availability Statement is currently as follows: “All relevant data are within the manuscript and its Supporting Information files.”

Please confirm at this time whether or not your submission contains all raw data required to replicate the results of your study. Authors must share the “minimal data set” for their submission. PLOS defines the minimal data set to consist of the data required to replicate all study findings reported in the article, as well as related metadata and methods (https://journals.plos.org/plosone/s/data-availability#loc-minimal-data-set-definition).

For example, authors should submit the following data:

- The values behind the means, standard deviations and other measures reported;

- The values used to build graphs;

- The points extracted from images for analysis.

Authors do not need to submit their entire data set if only a portion of the data was used in the reported study.

If your submission does not contain these data, please either upload them as Supporting Information files or deposit them to a stable, public repository and provide us with the relevant URLs, DOIs, or accession numbers. For a list of recommended repositories, please see https://journals.plos.org/plosone/s/recommended-repositories.

If there are ethical or legal restrictions on sharing a de-identified data set, please explain them in detail (e.g., data contain potentially sensitive information, data are owned by a third-party organization, etc.) and who has imposed them (e.g., an ethics committee). Please also provide contact information for a data access committee, ethics committee, or other institutional body to which data requests may be sent. If data are owned by a third party, please indicate how others may request data access.

Reply: The "Minimal Data Set Definition" file of the manuscript is available at: https://zenodo.org/records/20644876

4. If the reviewer comments include a recommendation to cite specific previously published works, please review and evaluate these publications to determine whether they are relevant and should be cited. There is no requirement to cite these works unless the editor has indicated otherwise.

Response: The suggested references have been verified and it has been confirmed that they are relevant to our work.

Additional Editor Comments:

1. The research questions and contribution are not sufficiently explicit. The introduction should end with clear research questions or objectives and explain how each methodological component addresses them. At present, the manuscript presents corpus construction, topic modelling, and intent classification, but the logical connection between these components and the study’s main research questions remains unclear.

Reply: Thank you very much for your comments. The research questions and the solutions, and the innovations of our work have been presented as follows:

**This study aims to address the following two core research questions:**

(1) how to build a large-scale high-quality corpus for the field of smart home and establish a reasonable intention tagging system to solve the problem of scarcity of domain corpus? (2) How to design an intention recognition model with high accuracy and interpretability, so that it not only outperforms the existing methods in classification performance, but also reveals the semantic structure of user intention from a data-driven perspective?

**The solution to the aforementioned questions:**

For the first research question, this study constructed a Chinese smart home corpus containing 120000 real conversations. The corpus uses a two-tier intention tagging framework (8 main categories and 42 subcategories) that fits the characteristics of the domain, which solves the problem of data missing from the source and provides a reliable basis for subsequent model training and evaluation. For the second research problem, this study uses the two-stage model architecture of "unsupervised topic discovery+supervised deep classification" to solve it. Firstly, the unsupervised topic recognition model is constructed by combining latent Dirichlet allocation (LDA) and K-means clustering (K-means) algorithm. From the perspective of data driven, the hidden semantic structure in the corpus is automatically mined, which is verified with the manual annotation system, so as to enhance the interpretability of the model; Secondly, a supervised intention classification model is constructed by fusing bidirectional encoder representations from transformers (BERT) and text convolutional neural network (textcnn), aiming to achieve high-precision intention recognition. Through the correspondence between the above two research questions and the corresponding methodological components one by one, the research systematically responds to the dual challenges of the scarcity of domain corpus and the lack of model interpretability.

**The objectives and innovations of our work:**

The research aims to systematically enhance the understanding ability of smart home dialogue systems by constructing domain specific corpora and designing high-performance interpretable intent recognition models. The innovation of the research lies in: (1) In theory, a two-stage intention recognition framework that integrates unsupervised topic discovery and supervised classification is proposed. Potential Dirichlet allocation and K-means clustering algorithm are introduced into smart home dialogue semantic mining, and the rationality of the intention annotation system is verified from a data-driven perspective. (2) Technically, a Chinese smart home corpus containing 120000 real conversations with a dual layer annotation structure was constructed, and a bidirectional transformer text convolutional neural network fusion model was designed, achieving a recognition accuracy of 96.7%. (3) In terms of application, quantitative analysis of user intention distribution from the perspective of data mining reveals high-frequency demand patterns centered on "device control" and "status query", providing practical data support for product function optimization.

2. The comparison with state-of-the-art methods is incomplete. The paper should include a clear comparison table covering recent intent classification and smart-home dialogue methods. The authors should justify why the selected baselines were chosen and explain whether stronger alternatives, such as transformer-only, CNN-only, BiLSTM/attention, or recent open-intent models, were considered.

Reply: Thank you very much for your comments. A comparison table has been added to justify the rationale for selecting specific baseline models. As follows:

To comprehensively evaluate the performance of the model, the study further compares it with a variety of recent advanced methods, including pure Bert classifier, pure textcnn and bilstm+attention mechanism. The selected baseline covers the mainstream technical route from traditional machine learning to pre training model, and can comprehensively test the comparative advantages of the proposed model. The overall performance comparison of each method on the test set is shown in Table 2.

Table 2 Comparison with advanced models

| Model | Accuracy | Macro F1 |
| --- | --- | --- |
| SVM | 89.50% | 89.20% |
| BiLSTM + Attention | 93.60% | 93.10% |
| TextCNN | 93.50% | 93.20% |
| BERT-base | 95.30% | 95.00% |
| BERT + BiLSTM + Attention | 95.80% | 95.50% |
| BERT-TextCNN | 96.70% | 96.40% |

It can be seen from table 2 that the proposed model performs best among all comparison methods. Compared with pure Bert, the accuracy of the fusion of textcnn is improved by 1.4%, indicating that the local n-gram feature has supplementary value for intention recognition Compared with bilstm+attention, it increased by 3.1%, which verified the advantages of the pre training model, and also showed that the closed intention classification architecture of this study was more suitable for smart home scenarios.

3. The methodological details are insufficient for reproducibility. The paper should clearly report model parameters, preprocessing steps, LDA settings, K-Means configuration, BERT/TextCNN architecture, training settings, validation strategy, and hyperparameter selection. PLOS ONE requires analyses to be described in sufficient detail and performed to a high technical standard.

Reply: Thank you very much for your comments. The methodological details have been provided, as follows:

**3.1 Experimental Settings**

The experiment uses the smart home discourse database constructed in Section 2.1, which contains 120000 dialogue sentences. According to the ratio of 8:1:1, the data set is randomly divided into training set, validation set and test set. The training set contains 96000 samples, the validation set contains 12000 samples, and the test set contains 12000 samples. The hierarchical sampling strategy is adopted to ensure that the distribution proportion of each intention category in the three subsets is consistent with the original data set. The training set is used for model parameter learning, the validation set is used for super parameter tuning and early stop decision, and the test set is only used for final performance evaluation, and does not participate in any training or tuning process.

In the data preprocessing stage, Jieba word segmentation tool is used for Chinese word segmentation, and a stop list containing 196 general stop words and 24 domain specific stop words is constructed at the same time. The maximum length of the text sequence is uniformly set to 128 characters. If it is insufficient, it will be filled in, and if it exceeds, it will be truncated. The LDA topic model sets the number of topics k=8, uses variational inference to estimate the parameters, sets the training rounds to 20, and sets the number of iterations per round to 200, and uses the automatic learning strategy for the super parameters α and η. The maximum number of TF-IDF feature extraction features is set to 5000, and the word items with document frequency less than 2 or more than 50% are filtered at the same time. The K-means clustering algorithm also sets the number of clusters to 8, uses the k-means++initialization strategy, the maximum number of iterations is 300, and the random seed is set to 42 to ensure that the results can be replicated. In the Bert textcnn model, Bert uses Bert base Chinese pre training weights, with 768 hidden layer dimensions, 12 multiple attention heads, and 12 transformer layers. Textcnn module uses three sizes of convolution cores, which are 2, 3 and 4 respectively. The number of convolution cores of each type is 100, and the convolution is followed by the global maximum pool operation. After the model fusion, the dropout layer is connected, and the discard rate is set to 0.5. Then it is connected to the full connection classification layer, and the output dimension is 42 (corresponding to the number of fine-grained intention categories). Adamw optimizer is used for model training. The initial learning rate is 2×10^-5^, the weight attenuation coefficient is 0.01, the batch size is 32, and the training rounds are 20. The learning rate adopts linear warm-up scheduling strategy, and the number of warm-up steps accounts for 10% of the total number of training steps. The loss function is the cross entropy loss, and the maximum norm of gradient clipping is set to 1.0. The verification strategy adopts early stopping method. If the macro average F1 score of the verification set has not been improved for three consecutive rounds, the training will be stopped and the best model weight will be restored. All experiments were run on a single NVIDIA Tesla V100 GPU (32GB video memory), with CUDA version of 11.7, pytorch version of 1.13.0, and transformers library version of 4.25.0.

4. Equations and technical formulas require proper formatting and citation. All equations should be numbered, consistently formatted, and cited when derived from previous work.

Reply: Thank you very much for your comments. The equation references in the paper have been verified and confirmed to be correct. A specific example is as follows:

$\left\{\begin{matrix} TF(t,d)=\frac{n_{t,d}}{\sum_{t^{'}\in d} n_{t^{'},d}} \\ IDF(t,D)=\log\frac{\left|D\right|}{\left|\left\{d\in D:t\in d\right\}\right|} \end{matrix}\right.$ (1)

In formula (1), $TF(t,d)$ signifies the word frequency of term $t$ in document $d$. $n_{t,d}$ signifies the number of times the $t$ appears in $d$. $\sum_{t^{'}\in d} n_{t^{'},d}$ signifies the total occurrences of all terms in $d$ [17].$\left|D\right|$ signifies the total documents in the corpus. $IDF(t,D)$ signifies the inverse document frequency of $t$. $\left|\left\{d\in D:t\in d\right\}\right|$ signifies the quantity of documents containing the $t$ [18].

[17]A. Ullah, S. M. Anwar, J. Li, L. Nadeem, T. Mahmood, A. Rehman and T. Saba. "Smart cities: The role of Internet of Things and machine learning in realizing a data-centric smart environment," Complex Intell. Syst., vol. 10, no. 1, pp. 1607–1637, February 2024, DOI:10.1007/s40747-023-01175-4.

[18]S. Pandey, S. Sharma and S. Wazir. "Mental healthcare chatbot based on natural language processing and deep learning approaches: Ted the therapist," Int. J. Inf. Technol., vol. 14, no. 7, pp. 3757–3766, July 2022, DOI:10.1007/s41870-022-00999-6.

5. Figures, tables, data, and code should follow PLOS ONE reporting standards. Figures should be revised according to PLOS ONE formatting expectations, and tables should be editable rather than image-based.

Reply: The reviewers' comments are acknowledged. It has been confirmed that all figures, tables, data, and code in the manuscript comply with the publication standards of PLOS ONE. The figures have been revised in accordance with the journal's formatting requirements, and the tables have been provided in editable formats. All content has been updated to meet the journal's requirements.

6. The authors should also provide access to the data or explain any restrictions, since PLOS requires data needed to replicate the study’s findings to be publicly available unless ethical or legal restrictions apply.

Reply: All data has been made publicly available.

Reviewer #1:

The paper presented an unsupervised topic discovery stage using LDA + K-Means with a supervised BERT-TextCNN classification model which is interesting but few changes in the paper are needed for further improving it and publishable standards. So I am choosing a minor revision. Here are my comments:

Strengths:

The paper has developed a big corpus for solving the problem with multiple data annotators. This maintains good quality in the annotations.

The contributions of the paper are interesting and important for the community.

The paper is well written and presented with very good diagrams and suitable tables

The paper is evaluated on multiple evaluation metrics.

Weaknesses:

The paper used 5 data annotators but the kappa scores are not mentioned. It is recommended to present the scores to understand the annotators agreement for the data annotation.

Reply: Thank you very much for your comments. An explanation of inter-annotator agreement (Kappa coefficient) has been added, as follows:

The tagging process consists of a tagging team composed of five trained taggers, using double-blind tagging and cross validation mechanisms. In order to evaluate the tagging consistency, the study randomly selected 10% of the corpus samples (about 8500 dialog sentences) for multiple tagging, and calculated the Fleiss' kappa coefficient between taggers. The calculation results show that the average Fleiss' kappa of the first level idea category is 0.847, and the average Fleiss' kappa of the second level fine-grained intention category is 0.802, both of which are at the "almost completely consistent" level, indicating that the two-tier annotation system has good reliability and operability. Regularly hold labeling seminars to solve ambiguity, and continuously update labeling guidelines to ensure the consistency of labeling [12].

The paper did not mention the train/val/test splits.

Reply: Thank you very much for your comments. The dataset split has been supplemented, as follows:

The experiment uses the smart home discourse database constructed in Section 2.1, which contains 120000 dialogue sentences. According to the ratio of 8:1:1, the data set is randomly divided into training set, validation set and test set. The training set contains 96000 samples, the validation set contains 12000 samples, and the test set contains 12000 samples. The hierarchical sampling strategy is adopted to ensure that the distribution proportion of each intention category in the three subsets is consistent with the original data set. The training set is used for model parameter learning, the validation set is used for super parameter tuning and early stop decision, and the test set is only used for final performance evaluation, and does not participate in any training or tuning process.

The limitations section is not presented in the paper. It is recommended to add as it will be presenting the gaps.

Reply: Thank you very much for your comments. A “Limitations and Future work” section has been added, as follows:

**5. Limitations and Future work**

This study has some limitations and needs to be further improved in future work. First of all, the corpus is mainly from Chinese. Although it covers the mainstream smart home interaction scene, it has not yet included dialect, Chinese English hybrid and multilingual dialogue data. The generalization ability of the model in non-standard Chinese expression still needs to be verified. Secondly, the current intention system is based on the closed world hypothesis, which assumes that all user inputs can be classified into 42 predefined intention categories, and the recognition ability of unknown intentions or new intention categories that do not appear in the training set has not been evaluated. Third, although the corpus contains 120000 conversations, it is mainly collected from public forums, device logs and simulated conversations. It lacks long-term continuous conversation data in a large-scale real home environment, and may not fully reflect the conversation context dependency in actual use. Fourth, the model training and evaluation are carried out on a single GPU server, and its reasoning efficiency has not been tested on edge computing devices or resource constrained embedded systems. The real-time performance of the actual deployment needs to be tested. Finally, the current model only supports intention recognition in a single round of conversation, and has not been extended to complex scenarios such as intention evolution tracking and context slot inheritance in multiple rounds of conversation. In the future, it can be further optimized by introducing a multilingual pre training model, enhancing the training of confrontation samples, building a larger cross scene corpus, and developing lightweight model compression technology.

The discussion should also present the error analysis it will help the researchers to understand where this model fails

Reply: Thank you very much for your comments. The error analysis has been provided, as follows:

To further understand the performance boundary of the model, the samples that were incorrectly classified in the test set were systematically analyzed. Statistics show that the overall error rate of the Bert textcnn model on the test set is 3.3%, and about 62% of the errors occur between intention categories with similar semantics. The most common confusion pairs include "temperature query" misjudged as "humidity query", "light brightness adjustment" misjudged as "light switch control" and "scene setting" misjudged as "mode switching". The analysis shows that such errors are mainly due to the lack of clear distinguishing keywords in user expression. Secondly, about 23% of the errors are related to complex sentence patterns, especially queries containing negative words or conditional clauses. The model performs poorly in dealing with such logical dependencies. Third, about 15% of the errors occurred in the intention category with less training samples. For example, the F1 scores of "system management" and "exception handling" were 93.6% and 92.9%, respectively, which were lower than the high-frequency intention category, indicating that the data imbalance had a negative impact on the performance of the model. The above analysis shows that the main failure modes of the current model focus on the differentiation of semantically similar intentions, the understanding of complex logical expressions, and the learning of low-frequency categories.

With the conclusion I strongly recommend the authors to present the future work as well. It helps to identify where the future work is going to be directed.

Reply: Thank you very much for your comments. The future work has been discussed in the “Limitations and Future work” section , as follows:

Looking forward to the future, the research will continue from the following directions. Firstly, in view of the confusion of the current model in the category of semantic similar intentions, it is planned to introduce a fine-grained comparative learning loss function to enhance the ability of the model to distinguish similar intentions. Secondly, aiming at the limitation of the lack of understanding ability of complex sentence patterns, this paper intends to explore the method of combining graph neural network with pre training model to explicitly model syntactic dependencies and logical structures. Third, in order to alleviate the impact of data imbalance on the performance of low-frequency categories, we will study the small sample learning strategy based on cue learning to reduce the dependence of the model on large-scale labeled data. Fourth, the current model only supports single round intention recognition. In the future, it will be extended to multiple rounds of conversation scenarios to develop a context aware intention tracking and slot inheritance module. Fifthly, facing the actual deployment requirements, it is planned to compress the current model through knowledge distillation and model quantification technology, so that it can run efficiently on edge devices such as smart speakers. Finally, a large-scale corpus containing multilingual, multilingual and real family continuous dialogues will be constructed to further enhance the generalization ability and ecological adaptability of the model.
