# Supplementary material for: Construction and optimization of deep learning intent classification model for smart home dialogue
Source: PLoS One. 2026 Sep 17;21(9):e0357708. doi: 10.1371/journal.pone.0357708 (PMC13585276; doi:10.1371/journal.pone.0357708)
Supplement: Supplementary file 3 [file pone.0357708.s003.doc]

PONE-D-26-09779R1

Construction and Optimization of Deep Learning Intent Classification Model for Smart Home Dialogue

PLOS One

Dear Dr. Lu,

Thank you for submitting your manuscript to PLOS ONE. After careful consideration, we feel that it has merit but does not fully meet PLOS ONE’s publication criteria as it currently stands. Therefore, we invite you to submit a revised version of the manuscript that addresses the points raised during the review process.

==============================

Kindly revisit and address all points raised by the Academic Editor, particularly those related to reproducibility. At present, the supporting materials appear insufficient: the manuscript references tables deposited in Zenodo, while the response letter points to a separate repository/location containing code embedded within Word documents. This format does not comply with PLOS ONE expectations for data and code availability and makes independent verification difficult.

Reply: The code has been re-provided and the format has been adjusted as per the requirements.

The "Code" files of the manuscript is available at: https://zenodo.org/records/21379700

In addition, the manuscript formatting and reporting still require substantial revision to meet PLOS ONE standards. The comparative evaluation should be strengthened by benchmarking against the most relevant state-of-the-art methods, with appropriate citations and direct comparisons to specific published models. Comparisons to broad model categories (e.g., "CNN" or "LSTM") are not adequate and do not allow readers to assess the true contribution of the proposed approach relative to the current literature.

Reply: Thank you very much for your suggestions. The format of the paper has been revised in accordance with the requirements of the journal. Meanwhile, comparisons with state-of-the-art methods have been added in the discussion section, as detailed below:

Finally, the research selects the most representative cutting-edge methods for smart home intention recognition task in recent years for comparison. In order to clearly show the performance differences of each model, table 3 summarizes the key indicators of the above frontier methods and the model proposed in this study in the task of smart home intention recognition.

Table 3 Performance comparison between the proposed model and state-of-the-art methods

| Model | Accuracy | F1-Score | Deployment Environment |
| --- | --- | --- | --- |
| GloVe-BiGRU-self-attention [29] | 89.72% | — | Server-side |
| Qwen2.5-0.5B (16-bit) [30] | 98.80% | — | Edge device |
| Qwen2.5-0.5B (4-bit quantized) [30] | 81.70% | — | Edge device |
| mBERT+Squeezeformer-CTC [31] | — | 91.50% | Resource-constrained device |
| BERT-TextCNN | 96.70% | 96.40% | Server-side |

Note:"—" indicates that the index is not involved in the quoted literature

It can be seen from table 3 that although the accuracy (98.8%) of the 16 bit large language model of birkmose et al. [30] is slightly higher than that of this study, the performance of its 4-bit quantized version has dropped sharply to 81.7%, indicating that the generative large language model faces a severe accuracy efficiency tradeoff when deployed at the edge, and the Bert textcnn model in this study avoids the performance loss caused by quantization with a lighter text coding architecture. Compared with the glove bigru self attention model of Li et al. [29], the accuracy of this study increased by nearly 7%, which verified the advantages of the pre training language model in smart home intention recognition. Compared with the 91.5% F1 score obtained by vysotska et al. [31] under the condition of limited resources, the macro average F1 score of this study reached 96.4%, which also showed obvious performance advantages. To sum up, the Bert textcnn model maintains high accuracy and robustness in closed intention classification tasks, and has better deployment flexibility than the generative large language model, which fully verifies its comprehensive advantages in practical applications.

Please carefully address these concerns and the issues previously raised by both the reviewers and the Academic Editor. We encourage the authors to take this opportunity to comprehensively strengthen the manuscript and ensure full compliance with PLOS ONE requirements.

==============================

**Journal Requirements:**

If the reviewer comments include a recommendation to cite specific previously published works, please review and evaluate these publications to determine whether they are relevant and should be cited. There is no requirement to cite these works unless the editor has indicated otherwise.

Reply: The reviewer did not suggest citing any references.

Reviewers' comments:

Reviewer's Responses to Questions

Comments to the Author

1. If the authors have adequately addressed your comments raised in a previous round of review and you feel that this manuscript is now acceptable for publication, you may indicate that here to bypass the “Comments to the Author” section, enter your conflict of interest statement in the “Confidential to Editor” section, and submit your "Accept" recommendation.

Reviewer #1: All comments have been addressed

Reply: Thank you very much for your satisfication with our revisions.

2. Is the manuscript technically sound, and do the data support the conclusions?

The manuscript must describe a technically sound piece of scientific research with data that supports the conclusions. Experiments must have been conducted rigorously, with appropriate controls, replication, and sample sizes. The conclusions must be drawn appropriately based on the data presented.

Reviewer #1: Yes

3. Has the statistical analysis been performed appropriately and rigorously?

Reviewer #1: Yes

4. Have the authors made all data underlying the findings in their manuscript fully available?

The PLOS Data policy requires authors to make all data underlying the findings described in their manuscript fully available without restriction, with rare exception (please refer to the Data Availability Statement in the manuscript PDF file). The data should be provided as part of the manuscript or its supporting information, or deposited to a public repository. For example, in addition to summary statistics, the data points behind means, medians and variance measures should be available. If there are restrictions on publicly sharing data—e.g. participant privacy or use of data from a third party—those must be specified.

Reviewer #1: Yes

5. Is the manuscript presented in an intelligible fashion and written in standard English?

PLOS ONE does not copyedit accepted manuscripts, so the language in submitted articles must be clear, correct, and unambiguous. Any typographical or grammatical errors should be corrected at revision, so please note any specific errors here.

Reviewer #1: Yes

6. Review Comments to the Author

Please use the space provided to explain your answers to the questions above. You may also include additional comments for the author, including concerns about dual publication, research ethics, or publication ethics. (Please upload your review as an attachment if it exceeds 20,000 characters)

Reviewer #1: (No Response)

Dear Dr. Lu,

We've checked your submission and before we can proceed, we need you to address the following issues:

1. We note that Figure 1 in your submission contain images which may be copyrighted. All PLOS content is published under the Creative Commons Attribution License (CC BY 4.0), which means that the manuscript, images, and Supporting Information files will be freely available online, and any third party is permitted to access, download, copy, distribute, and use these materials in any way, even commercially, with proper attribution.

We require you to either (1) present written permission from the copyright holder to publish these figures specifically under the CC BY 4.0 license, or (2) remove the figures from your submission:

1. You may seek permission from the original copyright holder of Figure 1 to publish the content specifically under the CC BY 4.0 license.

We recommend that you contact the original copyright holder with the Content Permission Form (http://journals.plos.org/plosone/s/file?id=7c09/content-permission-form.pdf) and the following text:

“I request permission for the open-access journal PLOS ONE to publish XXX under the Creative Commons Attribution License (CCAL) CC BY 4.0 (http://creativecommons.org/licenses/by/4.0/). Please be aware that this license allows unrestricted use and distribution, even commercially, by third parties. Please reply and provide explicit written permission to publish XXX under a CC BY license and complete the attached form.”

Please upload the completed Content Permission Form or other proof of granted permissions as an ""Other"" file with your submission.

In the figure caption of the copyrighted figure, please include the following text: “Reprinted from [ref] under a CC BY license, with permission from [name of publisher], original copyright [original copyright year].”

2. If you are unable to obtain permission from the original copyright holder to publish these figures under the CC BY 4.0 license or if the copyright holder’s requirements are incompatible with the CC BY 4.0 license, please either i) remove the figure or ii) supply a replacement figure that complies with the CC BY 4.0 license. Please check copyright information on all replacement figures and update the figure caption with source information. If applicable, please specify in the figure caption text when a figure is similar but not identical to the original image and is therefore for illustrative purposes only.

Reply: The suggestion is greatly appreciated. Since Figure 1 does not affect the overall readability of the paper, the final decision to delete Figure 1 has been made. The revised version is detailed in the manuscript.
