# Supplementary material for: Construction and optimization of deep learning intent classification model for smart home dialogue
Source: PLoS One. 2026 Sep 17;21(9):e0357708. doi: 10.1371/journal.pone.0357708 (PMC13585276; doi:10.1371/journal.pone.0357708)
Supplement: Supplementary file 4 [file pone.0357708.s005.doc]

PONE-D-26-09779R3

Several opportunities have been provided to address and resolve concerns regarding the reproducibility of the reported results. However, the materials submitted remain insufficient, consisting of poorly documented code and lacking the underlying data files required for independent verification. The data provided appear to be the final outputs used to generate the figures rather than the primary data and analysis pipeline needed to reproduce those results. You are welcome to submit a revised manuscript, provided that it fully complies with the PLOS ONE reproducibility requirements, including well-documented code, all necessary data files, and sufficient methodological detail to enable independent reproduction of the reported findings. Please note that this should be considered a final opportunity to address these concerns.

**Reply:** We sincerely thank the editor/reviewer for this critical comment and for providing opportunities to improve the reproducibility of our work. We fully recognize the previous shortcomings in our submitted materials, including insufficient code documentation and the absence of primary source data and full analysis pipelines for independent reproduction. We have comprehensively addressed all these concerns in this revised submission, strictly following PLOS ONE reproducibility requirements.

We have deposited the complete minimal reproducible dataset, full analysis pipeline, and custom source code into the Zenodo open‑access repository with persistent identifier: https://zenodo.org/records/21998257.

This repository contains:

De‑identified primary Chinese smart‑home dialogue corpus (original raw dialogue samples, train/validation/test split files, domain‑specific stop‑word list and annotation guidelines);

Full data preprocessing scripts;

Implementation of the unsupervised LDA‑K‑Means topic discovery model;

BERT‑TextCNN intent classification training, validation and evaluation source code;

Scripts for computing all metrics (NMI, ARI, Accuracy, Macro‑F1 etc.) and scripts that directly generate all figures and tables shown in the manuscript;

Comprehensive README documentation, software dependency list (requirements.txt), detailed step‑by‑step reproduction instructions, and MIT open‑source license. All random seeds are fixed to guarantee result reproducibility.

We have updated the Data Availability Statement of the manuscript to clearly cite the Zenodo repository link and describe the contents available in the archive.

We have supplemented additional text within the Experimental Settings subsection (Section 3.1) of Methods, explicitly referencing the repository and explaining how readers and reviewers can reproduce all experimental results reported in our paper.

Key documentation files from the Zenodo archive (README and dependency configuration file) are additionally uploaded as Supporting Information files within the submission system for convenient inspection.

With the above revisions, independent researchers can obtain primary input data, run the full analysis workflow, and reproduce all metrics, tables and figures presented in this manuscript. We believe the revised manuscript now fully satisfies PLOS ONE reproducibility standards.
