# Supplementary material for: Construction and optimization of deep learning intent classification model for smart home dialogue
Source: PLoS One. 2026 Sep 17;21(9):e0357708. doi: 10.1371/journal.pone.0357708 (PMC13585276; doi:10.1371/journal.pone.0357708)
Supplement: Peer review history

# Peer review: Decision Letter 0

**DOI:** 10.1371/journal.pone.0357708.r001

**Authors:** Issa Atoum

26 May 2026

Dear Dr. Lu,

Thank you for submitting your manuscript to PLOS ONE. After careful consideration, we feel that it has merit but does not fully meet PLOS ONE’s publication criteria as it currently stands. Therefore, we invite you to submit a revised version of the manuscript that addresses the points raised during the review process.

Please submit your revised manuscript by Jul 10 2026 11:59PM. If you will need more time than this to complete your revisions, please reply to this message or contact the journal office at plosone@plos.org. When you're ready to submit your revision, log on to https://www.editorialmanager.com/pone/ and select the 'Submissions Needing Revision' folder to locate your manuscript file.

- A letter that responds to each point raised by the academic editor and reviewer(s). You should upload this letter as a separate file labeled 'Response to Reviewers'.
- A marked-up copy of your manuscript that highlights changes made to the original version. You should upload this as a separate file labeled 'Revised Manuscript with Track Changes'.
- An unmarked version of your revised paper without tracked changes. You should upload this as a separate file labeled 'Manuscript'.

If you would like to make changes to your financial disclosure, please include your updated statement in your cover letter. Guidelines for resubmitting your figure files are available below the reviewer comments at the end of this letter.

If applicable, we recommend that you deposit your laboratory protocols in protocols.io to enhance the reproducibility of your results. Protocols.io assigns your protocol its own identifier (DOI) so that it can be cited independently in the future. For instructions see: https://journals.plos.org/plosone/s/submission-guidelines#loc-laboratory-protocols. Additionally, PLOS ONE offers an option for publishing peer-reviewed Lab Protocol articles, which describe protocols hosted on protocols.io. Read more information on sharing protocols at https://plos.org/protocols?utm_medium=editorial-email&utm_source=authorletters&utm_campaign=protocols.

As the corresponding author, your ORCID iD is verified in the submission system and will appear in the published article. PLOS supports the use of ORCID, and we encourage all coauthors to register for an ORCID iD and use it as well. Please encourage your coauthors to verify their ORCID iD within the submission system before final acceptance, as unverified ORCID iDs will not appear in the published article. *Only*  the individual author can complete the verification step; PLOS staff *cannot*  verify ORCID iDs on behalf of authors.

We look forward to receiving your revised manuscript.

Kind regards,

Issa Atoum

Academic Editor

PLOS One

Journal Requirements:

When submitting your revision, we need you to address these additional requirements.

1.Please ensure that your manuscript meets PLOS ONE's style requirements, including those for file naming. The PLOS ONE style templates can be found at

https://journals.plos.org/plosone/s/file?id=wjVg/PLOSOne_formatting_sample_main_body.pdf and

https://journals.plos.org/plosone/s/file?id=ba62/PLOSOne_formatting_sample_title_authors_affiliations.pdf

2. Please note that PLOS One has specific guidelines on code sharing for submissions in which author-generated code underpins the findings in the manuscript. In these cases, we expect all author-generated code to be made available without restrictions upon publication of the work. Please review our guidelines at https://journals.plos.org/plosone/s/materials-and-software-sharing#loc-sharing-code and ensure that your code is shared in a way that follows best practice and facilitates reproducibility and reuse.

3. We note that your Data Availability Statement is currently as follows: “All relevant data are within the manuscript and its Supporting Information files.”

Please confirm at this time whether or not your submission contains all raw data required to replicate the results of your study. Authors must share the “minimal data set” for their submission. PLOS defines the minimal data set to consist of the data required to replicate all study findings reported in the article, as well as related metadata and methods (https://journals.plos.org/plosone/s/data-availability#loc-minimal-data-set-definition).

For example, authors should submit the following data:

- The values behind the means, standard deviations and other measures reported;

- The values used to build graphs;

- The points extracted from images for analysis.

Authors do not need to submit their entire data set if only a portion of the data was used in the reported study.

If your submission does not contain these data, please either upload them as Supporting Information files or deposit them to a stable, public repository and provide us with the relevant URLs, DOIs, or accession numbers. For a list of recommended repositories, please see https://journals.plos.org/plosone/s/recommended-repositories.

If there are ethical or legal restrictions on sharing a de-identified data set, please explain them in detail (e.g., data contain potentially sensitive information, data are owned by a third-party organization, etc.) and who has imposed them (e.g., an ethics committee). Please also provide contact information for a data access committee, ethics committee, or other institutional body to which data requests may be sent. If data are owned by a third party, please indicate how others may request data access.

4. If the reviewer comments include a recommendation to cite specific previously published works, please review and evaluate these publications to determine whether they are relevant and should be cited. There is no requirement to cite these works unless the editor has indicated otherwise.

Additional Editor Comments:

1. The research questions and contribution are not sufficiently explicit. The introduction should end with clear research questions or objectives and explain how each methodological component addresses them. At present, the manuscript presents corpus construction, topic modelling, and intent classification, but the logical connection between these components and the study’s main research questions remains unclear.

2. The comparison with state-of-the-art methods is incomplete. The paper should include a clear comparison table covering recent intent classification and smart-home dialogue methods. The authors should justify why the selected baselines were chosen and explain whether stronger alternatives, such as transformer-only, CNN-only, BiLSTM/attention, or recent open-intent models, were considered.

3. The methodological details are insufficient for reproducibility. The paper should clearly report model parameters, preprocessing steps, LDA settings, K-Means configuration, BERT/TextCNN architecture, training settings, validation strategy, and hyperparameter selection. PLOS ONE requires analyses to be described in sufficient detail and performed to a high technical standard.

4. Equations and technical formulas require proper formatting and citation. All equations should be numbered, consistently formatted, and cited when derived from previous work.

5. Figures, tables, data, and code should follow PLOS ONE reporting standards. Figures should be revised according to PLOS ONE formatting expectations, and tables should be editable rather than image-based.

6. The authors should also provide access to the data or explain any restrictions, since PLOS requires data needed to replicate the study’s findings to be publicly available unless ethical or legal restrictions apply.

[Note: HTML markup is below. Please do not edit.]

Reviewer's Responses to Questions

**Comments to the Author**

1. Is the manuscript technically sound, and do the data support the conclusions?

Reviewer #1: Yes

**********

2. Has the statistical analysis been performed appropriately and rigorously?

Reviewer #1: Yes

**********

3. Have the authors made all data underlying the findings in their manuscript fully available?

The PLOS Data policy

Reviewer #1: Yes

**********

4. Is the manuscript presented in an intelligible fashion and written in standard English?

Reviewer #1: Yes

**********

Reviewer #1: The paper presented an unsupervised topic discovery stage using LDA + K-Means with a supervised BERT-TextCNN classification model which is interesting but few changes in the paper are needed for further improving it and publishable standards. So I am choosing a minor revision. Here are my comments:

Strengths:

The paper has developed a big corpus for solving the problem with multiple data annotators. This maintains good quality in the annotations.

The contributions of the paper are interesting and important for the community.

The paper is well written and presented with very good diagrams and suitable tables

The paper is evaluated on multiple evaluation metrics.

Weaknesses:

The paper used 5 data annotators but the kappa scores are not mentioned. It is recommended to present the scores to understand the annotators agreement for the data annotation.

The paper did not mention the train/val/test splits.

The limitations section is not presented in the paper. It is recommended to add as it will be presenting the gaps.

The discussion should also present the error analysis it will help the researchers to understand where this model fails

With the conclusion I strongly recommend the authors to present the future work as well. It helps to identify where the future work is going to be directed.

**********

what does this mean?). If published, this will include your full peer review and any attached files.

If you choose “no”, your identity will remain anonymous but your review may still be made public.

**Do you want your identity to be public for this peer review?**  For information about this choice, including consent withdrawal, please see our Privacy Policy

Reviewer #1: **Yes:**  Karthik Ramamurthy

**********

[NOTE: If reviewer comments were submitted as an attachment file, they will be attached to this email and accessible via the submission site. Please log into your account, locate the manuscript record, and check for the action link "View Attachments". If this link does not appear, there are no attachment files.]

To ensure your figures meet our technical requirements, please review our figure guidelines: https://journals.plos.org/plosone/s/figures

You may also use PLOS’s free figure tool, NAAS, to help you prepare publication quality figures: https://journals.plos.org/plosone/s/figures#loc-tools-for-figure-preparation.

NAAS will assess whether your figures meet our technical requirements by comparing each figure against our figure specifications.

# Author comment: Author response to Decision Letter 1

**DOI:** 10.1371/journal.pone.0357708.r002

11 Jun 2026

The manuscript has been revised according to comments.

Attachment Submitted filename: Responsed to comments.doc

# Peer review: Decision Letter 1

**DOI:** 10.1371/journal.pone.0357708.r003

**Authors:** Issa Atoum

6 Jul 2026

Dear Dr. Lu,

Thank you for submitting your manuscript to PLOS ONE. After careful consideration, we feel that it has merit but does not fully meet PLOS ONE’s publication criteria as it currently stands. Therefore, we invite you to submit a revised version of the manuscript that addresses the points raised during the review process.

Kindly revisit and address all points raised by the Academic Editor, particularly those related to reproducibility. At present, the supporting materials appear insufficient: the manuscript references tables deposited in Zenodo, while the response letter points to a separate repository/location containing code embedded within Word documents. This format does not comply with PLOS ONE expectations for data and code availability and makes independent verification difficult.

In addition, the manuscript formatting and reporting still require substantial revision to meet PLOS ONE standards. The comparative evaluation should be strengthened by benchmarking against the most relevant state-of-the-art methods, with appropriate citations and direct comparisons to specific published models. Comparisons to broad model categories (e.g., "CNN" or "LSTM") are not adequate and do not allow readers to assess the true contribution of the proposed approach relative to the current literature.

Please carefully address these concerns and the issues previously raised by both the reviewers and the Academic Editor. We encourage the authors to take this opportunity to comprehensively strengthen the manuscript and ensure full compliance with PLOS ONE requirements.

Please submit your revised manuscript by Aug 20 2026 11:59PM. If you will need more time than this to complete your revisions, please reply to this message or contact the journal office at plosone@plos.org. When you're ready to submit your revision, log on to https://www.editorialmanager.com/pone/ and select the 'Submissions Needing Revision' folder to locate your manuscript file.

- A letter that responds to each point raised by the academic editor and reviewer(s). You should upload this letter as a separate file labeled 'Response to Reviewers'.
- A marked-up copy of your manuscript that highlights changes made to the original version. You should upload this as a separate file labeled 'Revised Manuscript with Track Changes'.
- An unmarked version of your revised paper without tracked changes. You should upload this as a separate file labeled 'Manuscript'.

If you would like to make changes to your financial disclosure, please include your updated statement in your cover letter. Guidelines for resubmitting your figure files are available below the reviewer comments at the end of this letter.

If applicable, we recommend that you deposit your laboratory protocols in protocols.io to enhance the reproducibility of your results. Protocols.io assigns your protocol its own identifier (DOI) so that it can be cited independently in the future. For instructions see: https://journals.plos.org/plosone/s/submission-guidelines#loc-laboratory-protocols. Additionally, PLOS ONE offers an option for publishing peer-reviewed Lab Protocol articles, which describe protocols hosted on protocols.io. Read more information on sharing protocols at https://plos.org/protocols?utm_medium=editorial-email&utm_source=authorletters&utm_campaign=protocols.

As the corresponding author, your ORCID iD is verified in the submission system and will appear in the published article. PLOS supports the use of ORCID, and we encourage all coauthors to register for an ORCID iD and use it as well. Please encourage your coauthors to verify their ORCID iD within the submission system before final acceptance, as unverified ORCID iDs will not appear in the published article. *Only*  the individual author can complete the verification step; PLOS staff *cannot*  verify ORCID iDs on behalf of authors.

We look forward to receiving your revised manuscript.

Kind regards,

Issa Atoum

Academic Editor

PLOS One

Journal Requirements:

If the reviewer comments include a recommendation to cite specific previously published works, please review and evaluate these publications to determine whether they are relevant and should be cited. There is no requirement to cite these works unless the editor has indicated otherwise.

[Note: HTML markup is below. Please do not edit.]

Reviewers' comments:

Reviewer's Responses to Questions

**Comments to the Author**

Reviewer #1: All comments have been addressed

**********

2. Is the manuscript technically sound, and do the data support the conclusions?

Reviewer #1: Yes

**********

3. Has the statistical analysis been performed appropriately and rigorously?

Reviewer #1: Yes

**********

4. Have the authors made all data underlying the findings in their manuscript fully available?

The PLOS Data policy

Reviewer #1: Yes

**********

5. Is the manuscript presented in an intelligible fashion and written in standard English?

Reviewer #1: Yes

**********

Reviewer #1: (No Response)

**********

what does this mean?). If published, this will include your full peer review and any attached files.

If you choose “no”, your identity will remain anonymous but your review may still be made public.

**Do you want your identity to be public for this peer review?**  For information about this choice, including consent withdrawal, please see our Privacy Policy

Reviewer #1: **Yes:**  Karthik Ramamurthy

**********

[NOTE: If reviewer comments were submitted as an attachment file, they will be attached to this email and accessible via the submission site. Please log into your account, locate the manuscript record, and check for the action link "View Attachments". If this link does not appear, there are no attachment files.]

To ensure your figures meet our technical requirements, please review our figure guidelines: https://journals.plos.org/plosone/s/figures

You may also use PLOS’s free figure tool, NAAS, to help you prepare publication quality figures: https://journals.plos.org/plosone/s/figures#loc-tools-for-figure-preparation.

NAAS will assess whether your figures meet our technical requirements by comparing each figure against our figure specifications.

# Author comment: Author response to Decision Letter 2

**DOI:** 10.1371/journal.pone.0357708.r004

28 Jul 2026

The manuscript has been revised according to comments.

Attachment Submitted filename: Responsed_to_comments_auresp_2.doc

# Editor report: Decision Letter 2

**DOI:** 10.1371/journal.pone.0357708.r005

**Authors:** Issa Atoum

30 Jul 2026

Dear Dr. Lu,

Thank you for submitting your manuscript to PLOS ONE. After careful consideration, we feel that it has merit but does not fully meet PLOS ONE’s publication criteria as it currently stands. Therefore, we invite you to submit a revised version of the manuscript that addresses the points raised during the review process.

Please submit your revised manuscript by Sep 13 2026 11:59PM. If you will need more time than this to complete your revisions, please reply to this message or contact the journal office at plosone@plos.org. When you're ready to submit your revision, log on to https://www.editorialmanager.com/pone/ and select the 'Submissions Needing Revision' folder to locate your manuscript file.

- A letter that responds to each point raised by the academic editor and reviewer(s). You should upload this letter as a separate file labeled 'Response to Reviewers'.
- A marked-up copy of your manuscript that highlights changes made to the original version. You should upload this as a separate file labeled 'Revised Manuscript with Track Changes'.
- An unmarked version of your revised paper without tracked changes. You should upload this as a separate file labeled 'Manuscript'.

If you would like to make changes to your financial disclosure, please include your updated statement in your cover letter. Guidelines for resubmitting your figure files are available below the reviewer comments at the end of this letter.

If applicable, we recommend that you deposit your laboratory protocols in protocols.io to enhance the reproducibility of your results. Protocols.io assigns your protocol its own identifier (DOI) so that it can be cited independently in the future. For instructions see: https://journals.plos.org/plosone/s/submission-guidelines#loc-laboratory-protocols. Additionally, PLOS ONE offers an option for publishing peer-reviewed Lab Protocol articles, which describe protocols hosted on protocols.io. Read more information on sharing protocols at https://plos.org/protocols?utm_medium=editorial-email&utm_source=authorletters&utm_campaign=protocols.

As the corresponding author, your ORCID iD is verified in the submission system and will appear in the published article. PLOS supports the use of ORCID, and we encourage all coauthors to register for an ORCID iD and use it as well. Please encourage your coauthors to verify their ORCID iD within the submission system before final acceptance, as unverified ORCID iDs will not appear in the published article. *Only*  the individual author can complete the verification step; PLOS staff *cannot*  verify ORCID iDs on behalf of authors.

We look forward to receiving your revised manuscript.

Kind regards,

Issa Atoum

Academic Editor

PLOS One

Journal Requirements:

If the reviewer comments include a recommendation to cite specific previously published works, please review and evaluate these publications to determine whether they are relevant and should be cited. There is no requirement to cite these works unless the editor has indicated otherwise.

[Note: HTML markup is below. Please do not edit.]

[NOTE: If reviewer comments were submitted as an attachment file, they will be attached to this email and accessible via the submission site. Please log into your account, locate the manuscript record, and check for the action link "View Attachments". If this link does not appear, there are no attachment files.]

To ensure your figures meet our technical requirements, please review our figure guidelines: https://journals.plos.org/plosone/s/figures

You may also use PLOS’s free figure tool, NAAS, to help you prepare publication quality figures: https://journals.plos.org/plosone/s/figures#loc-tools-for-figure-preparation.

NAAS will assess whether your figures meet our technical requirements by comparing each figure against our figure specifications.

# Author comment: Author response to Decision Letter 3

**DOI:** 10.1371/journal.pone.0357708.r006

7 Aug 2026

The manuscript has been revised according to comments.

Attachment Submitted filename: Responsed to comments.docx

# Editor report: Decision Letter 3

**DOI:** 10.1371/journal.pone.0357708.r007

**Authors:** Issa Atoum

11 Aug 2026

Dear Dr. Lu,

Thank you for submitting your manuscript to PLOS ONE. After careful consideration, we feel that it has merit but does not fully meet PLOS ONE’s publication criteria as it currently stands. Therefore, we invite you to submit a revised version of the manuscript that addresses the points raised during the review process.

==============================

Several opportunities have been provided to address and resolve concerns regarding the reproducibility of the reported results. However, the materials submitted remain insufficient, consisting of poorly documented code and lacking the underlying data files required for independent verification. The data provided appear to be the final outputs used to generate the figures rather than the primary data and analysis pipeline needed to reproduce those results. You are welcome to submit a revised manuscript, provided that it fully complies with the PLOS ONE reproducibility requirements, including well-documented code, all necessary data files, and sufficient methodological detail to enable independent reproduction of the reported findings. Please note that this should be considered a final opportunity to address these concerns.

==============================

Please submit your revised manuscript by Sep 25 2026 11:59PM. If you will need more time than this to complete your revisions, please reply to this message or contact the journal office at plosone@plos.org. When you're ready to submit your revision, log on to https://www.editorialmanager.com/pone/ and select the 'Submissions Needing Revision' folder to locate your manuscript file.

- A letter that responds to each point raised by the academic editor and reviewer(s). You should upload this letter as a separate file labeled 'Response to Reviewers'.
- A marked-up copy of your manuscript that highlights changes made to the original version. You should upload this as a separate file labeled 'Revised Manuscript with Track Changes'.
- An unmarked version of your revised paper without tracked changes. You should upload this as a separate file labeled 'Manuscript'.

If you would like to make changes to your financial disclosure, please include your updated statement in your cover letter. Guidelines for resubmitting your figure files are available below the reviewer comments at the end of this letter.

If applicable, we recommend that you deposit your laboratory protocols in protocols.io to enhance the reproducibility of your results. Protocols.io assigns your protocol its own identifier (DOI) so that it can be cited independently in the future. For instructions see: https://journals.plos.org/plosone/s/submission-guidelines#loc-laboratory-protocols. Additionally, PLOS ONE offers an option for publishing peer-reviewed Lab Protocol articles, which describe protocols hosted on protocols.io. Read more information on sharing protocols at https://plos.org/protocols?utm_medium=editorial-email&utm_source=authorletters&utm_campaign=protocols.

As the corresponding author, your ORCID iD is verified in the submission system and will appear in the published article. PLOS supports the use of ORCID, and we encourage all coauthors to register for an ORCID iD and use it as well. Please encourage your coauthors to verify their ORCID iD within the submission system before final acceptance, as unverified ORCID iDs will not appear in the published article. *Only*  the individual author can complete the verification step; PLOS staff *cannot*  verify ORCID iDs on behalf of authors.

We look forward to receiving your revised manuscript.

Kind regards,

Issa Atoum

Academic Editor

PLOS One

Journal Requirements:

If the reviewer comments include a recommendation to cite specific previously published works, please review and evaluate these publications to determine whether they are relevant and should be cited. There is no requirement to cite these works unless the editor has indicated otherwise.

[Note: HTML markup is below. Please do not edit.]

[NOTE: If reviewer comments were submitted as an attachment file, they will be attached to this email and accessible via the submission site. Please log into your account, locate the manuscript record, and check for the action link "View Attachments". If this link does not appear, there are no attachment files.]

To ensure your figures meet our technical requirements, please review our figure guidelines: https://journals.plos.org/plosone/s/figures

You may also use PLOS’s free figure tool, NAAS, to help you prepare publication quality figures: https://journals.plos.org/plosone/s/figures#loc-tools-for-figure-preparation.

NAAS will assess whether your figures meet our technical requirements by comparing each figure against our figure specifications.

# Author comment: Author response to Decision Letter 4

**DOI:** 10.1371/journal.pone.0357708.r008

19 Aug 2026

The manuscript has been revised according to comments.

Attachment Submitted filename: Responsed_to_comments_auresp_4.doc

# Editor report: Decision Letter 4

**DOI:** 10.1371/journal.pone.0357708.r009

**Authors:** Issa Atoum

22 Aug 2026

Construction and Optimization of Deep Learning Intent Classification Model for Smart Home Dialogue

PONE-D-26-09779R4

Dear Dr. Lu,

We’re pleased to inform you that your manuscript has been judged scientifically suitable for publication and will be formally accepted for publication once it meets all outstanding technical requirements.

Within one week, you’ll receive an e-mail detailing the required amendments. When these have been addressed, you’ll receive a formal acceptance letter and your manuscript will be scheduled for publication.

An invoice will be generated when your article is formally accepted. Please note, if your institution has a publishing partnership with PLOS and your article meets the relevant criteria, all or part of your publication costs will be covered. Please make sure your user information is up-to-date by logging into Editorial Manager at Editorial Manager® and clicking the ‘Update My Information' link at the top of the page. For questions related to billing, please contact billing support.

If your institution or institutions have a press office, please notify them about your upcoming paper to help maximize its impact. If they’ll be preparing press materials, please inform our press team as soon as possible -- no later than 48 hours after receiving the formal acceptance. Your manuscript will remain under strict press embargo until 2 pm Eastern Time on the date of publication. For more information, please contact onepress@plos.org.

Kind regards,

Issa Atoum

Academic Editor

PLOS One

Additional Editor Comments (optional):

Reviewers' comments:

# Editor report: Acceptance letter

**DOI:** 10.1371/journal.pone.0357708.r010

**Authors:** Issa Atoum

PONE-D-26-09779R4

PLOS One

Dear Dr. Lu,

I'm pleased to inform you that your manuscript has been deemed suitable for publication in PLOS One. Congratulations! Your manuscript is now being handed over to our production team.

At this stage, our production department will prepare your paper for publication. This includes ensuring the following:

* All references, tables, and figures are properly cited

* All relevant supporting information is included in the manuscript submission,

* There are no issues that prevent the paper from being properly typeset

You will receive further instructions from the production team, including instructions on how to review your proof when it is ready. Please keep in mind that we are working through a large volume of accepted articles, so please give us a few days to review your paper and let you know the next and final steps.

Lastly, if your institution or institutions have a press office, please let them know about your upcoming paper now to help maximize its impact. If they'll be preparing press materials, please inform our press team within the next 48 hours. Your manuscript will remain under strict press embargo until 2 pm Eastern Time on the date of publication. For more information, please contact onepress@plos.org.

You will receive an invoice from PLOS for your publication fee after your manuscript has reached the completed accept phase. If you receive an email requesting payment before acceptance or for any other service, this may be a phishing scheme. Learn how to identify phishing emails and protect your accounts at https://explore.plos.org/phishing.

If we can help with anything else, please email us at customercare@plos.org.

Thank you for submitting your work to PLOS One and supporting open access.

Kind regards,

PLOS One Editorial Office Staff

on behalf of

Dr. Issa Atoum

Academic Editor

PLOS One
